# Safety Assessment of Newly Expressed Proteins in Genetically Modified Crops: A Decade of Regulatory Case Studies

**DOI:** 10.3390/plants15213343

**Published:** 2026-10-28

**Authors:** Cunxi Wang, Christopher R. Brown, Tommi A. White, Beverly F. Anderson, Yong Yin, Harit K. Bal, Collin J. Preftakes, Rong Wang, Scott A. Saracco, Kimberly Hodge-Bell, Steven L. Levine, David Caldwell

**Affiliations:** 1 Regulatory Science, Bayer Crop Science, Chesterfield, MO 63017, USA; christopher.brown1@bayer.com (C.R.B.); beverly.anderson1@bayer.com (B.F.A.); yong.yin@bayer.com (Y.Y.); haritkaur.bal@bayer.com (H.K.B.); collin.preftakes@bayer.com (C.J.P.); rong.wang@bayer.com (R.W.); scott.saracco@bayer.com (S.A.S.); kimberly.hodge-bell@bayer.com (K.H.-B.); steven.levine1@bayer.com (S.L.L.); david.caldwell@bayer.com (D.C.); 2 Plant Biotechnology, Bayer Crop Science, Chesterfield, MO 63017, USA; tommi.white@bayer.com

**Keywords:** genetically modified crops, newly expressed proteins, protein safety assessment, ecological risk assessment, bridging approach

## Abstract

This review synthesizes regulatory and scientific evidence accumulated over the past decade supporting the safety of newly expressed proteins (NEPs) in genetically modified (GM) crops. Using five case studies representing diverse protein classes, including dicamba monooxygenase (DMO), protoporphyrinogen IX oxidase (PPO), Cry1B.2/Cry1B.3, Mpp75Aa1.1, and Vip3Cb1, it illustrates how science-based, case-by-case, weight-of-evidence approaches are applied in food and feed safety assessment and ecological risk assessment (ERA) for regulatory submissions. These examples demonstrate how hazard identification, hazard characterization, and exposure assessment are integrated across proteins with diverse biological functions, structural characteristics, and levels of familiarity. It further examines the safety relevance of protein processing and sequence variation arising from protein engineering or biological processing and discusses the scientific basis supporting bridging and read-across approaches, including phylogenetic relationships, sequence similarity, structural conservation, domain architecture, and functional equivalence. The increasing application of safe-by-design principles and bridging strategies is highlighted as an important advancement enabling efficient, scientifically robust evaluations while reducing unnecessary testing. The review also discusses the convergence of food/feed safety assessment and ERA and future directions. Overall, accumulated evidence from NEPs commercialized to date in GM crops has not identified hazards for the assessed safety endpoints and supports the continued evolution and modernization of protein safety assessment frameworks.

## 1. Introduction

As global food demand continues to increase, modern farmers face persistent challenges including insect pest pressure and weed management that impact yield [1,2,3]. Genetically modified (GM) crops have become a well-established and widely adopted agricultural technology to help address these challenges. A substantial body of evidence demonstrates that GM crop adoption is associated with improved agronomic and economic outcomes including higher yields, increased farm income, and reduced input costs, contributing to more sustainable production systems [4,5,6,7,8]. Since their commercial introduction in 1996, GM crops have expanded globally, with more than 650 distinct transgenic events receiving regulatory approval for cultivation and/or import use in at least one country or region, spanning eight major trait categories, including insect resistance, herbicide tolerance, product quality enhancement, and other agronomic or consumer-benefit traits, and approved across 46 countries or regions [9].

Before commercialization, all newly expressed proteins (NEPs) in GM crops undergo comprehensive assessments of food and feed safety, as well as evaluations of potential environmental impacts, to support regulatory approval in each region. Potential food and feed safety risks of NEPs are evaluated by integrating multiple lines of evidence relevant to toxicity and allergenicity. In cases where the GM product is intended for environmental release (e.g., cultivation countries), ecological risk assessments (ERAs) are conducted and potential environmental impacts of NEPs are evaluated where the likelihood of harm to valued ecological entities (e.g., biodiversity, ecosystem services) is assessed by integrating information on both hazard and exposure.

NEP regulatory assessments for food and feed safety are conducted within internationally recognized frameworks established by the FAO (Food and Agriculture Organization)/WHO (World Health Organization) Codex Alimentarius Commission [10]. Over the past three decades, these Codex frameworks have been effectively applied to all protein classes including those that confer insect resistance, herbicide tolerance, and yield and stress improvements that form the basis of most commercialized traits [9]. ERAs for NEPs are generally focused on insect resistance traits in countries where cultivation of the GM crop is anticipated. The ERA framework is based on regulatory publications, consensus documents, and peer-reviewed publications [11,12,13,14]. NEP ERAs are conducted on a case-by-case basis through careful consideration of potential pathways to harm based on characteristics of the NEP, crop, and receiving environment. The accumulation of knowledge from these safety assessments for approved events has established a robust weight-of-evidence for the safety of NEPs and has presented opportunities for streamlining and simplification of ERA data requirements without compromising the integrity of the safety assessment. At the same time, opportunities remain to further harmonize regulatory implementation and acceptance of emerging scientific approaches across regulatory systems.

Over the last decade, regulatory frameworks for food and feed safety assessments have increasingly incorporated the principles of the 3Rs (Replacement, Reduction, and Refinement), to minimize animal use while maintaining scientific rigor and ensuring that risk management objectives are met [15,16,17,18]. Similarly, data streamlining opportunities and increased use of scientific rationale in lieu of study data for NEP ERAs reflect extensive ERA experience with GM crops over the past 30 years [19]. This evolution aligns with a broader shift toward more efficient, evidence-based, and risk-proportionate assessment strategies based on the extensive experience in GM crop regulation accumulated over the years.

More recently, advances in plant biotechnology have expanded the diversity of proteins introduced into GM crops. From the food and feed safety perspective, these proteins can be broadly categorized into two groups: (1) those with structural and functional similarity to previously assessed proteins or with established familiarity to well-characterized or dietary proteins, and (2) novel proteins that exhibit limited similarity to proteins with known safety profiles [20]. This diversification raises a key question: how can existing safety assessment frameworks be applied consistently and proportionately across proteins with varying degrees of familiarity?

For proteins within the first category, established frameworks provide a basis for leveraging accumulated knowledge, comparative analyses, and prior safety data to support efficient and scientifically robust evaluations consistent with the 3Rs principles. In such cases, safety conclusions from a similar protein may be used to evaluate a NEP and therefore reduce the need for additional studies, including animal-based studies. In contrast, proteins in the second category may require targeted assessments to address any unique characteristics, particularly where traditional testing approaches are less applicable [15,21]. Across both categories, safe-by-design principles are integrated throughout product development. NEPs with potential food/feed or environmental hazards (i.e., those with potential structural and functional similarity to known toxins or allergens) are eliminated from advancement in the product pipeline. This balance is critical to maintaining both scientific robustness and regulatory consistency while enabling timely market access of GM crop innovations.

Likewise, the diversification of proteins used in GM crops has raised new questions for ERAs. At the same time as data reductions are considered based on the long history of GM crop ERA for familiar proteins, NEPs with structural diversity from new source organisms or expanded activity spectra have raised the question of whether the traditional ERA framework can accommodate the novel proteins. Over the last decade, ERAs have tested the ability of the ERA framework to effectively assess NEPs for insect resistance traits.

Recent scientific and regulatory discussions, including EFSA-led initiatives, highlight opportunities to refine food and feed safety assessment frameworks through the integration of new approach methodologies (NAMs) within a tiered, weight-of-evidence paradigm that prioritizes in silico and in vitro evidence [20]. These approaches are supported by structured concepts such as history of safe use (HoSU), familiarity, and read-across, and bridging strategies that facilitate data transportability by enabling relevant evidence to be leveraged across related proteins. In parallel, these new in silico methods require the development of harmonized and curated comparator databases, encompassing well-characterized proteins and toxin/allergen reference datasets to support robust computational screening and transparent decision-making. Collectively, these advances further align with the 3Rs principles while enhancing the consistency and efficiency of safety assessments.

Similarly, pragmatic bridging approaches have been increasingly leveraged for ERAs where data generated for an insecticidal protein are later used to support the ERA of GM crops expressing a similar protein [22,23]. The concept of data transportability has recently been further applied to ERA by using non-target organism (NTO) field data generated in one country to support the ERA in another country and thus avoiding unnecessary repetition of studies [24]. The long HoSU of GM crops adds confidence to the use of bridging approaches and data transportability.

Within this evolving technological and regulatory landscape, the objective of this review is to synthesize case study–driven evidence from the past decade demonstrating how contemporary food and feed safety and ERA frameworks have been applied across diverse classes of NEPs. Drawing on real-world regulatory assessments spanning human, animal, and environmental considerations, it highlights how foundational principles, such as safe-by-design, source organism familiarity, protein familiarity, function, digestibility, toxicity, and ecological relevance, remain robust and adaptable. In addition, the review discusses the growing role of protein-based, weight-of-evidence assessment frameworks, including bridging and read-across as well as data transportability approaches, in supporting the evolution of safety assessment of NEPs by leveraging existing knowledge and data to enable more efficient, hypothesis-driven decision-making. Together, these insights underscore the continued advancement of protein safety evaluation toward a scientifically grounded, weight-of-evidence, and safe-by-design paradigm supporting future biotechnological innovation.

## 2. Core Principles of Protein Safety Assessment: A Safe-by-Design, Weight-of-Evidence Framework

The assessment of NEPs begins with problem formulation to define relevant assessment questions, relevant exposure scenarios, and hazard hypotheses that inform the weight-of-evidence evaluation. Risk is characterized through a weight-of-evidence approach by integrating hazard and exposure information. For food and feed safety, this weight-of-evidence is built through combined information on HoSU of the protein or donor organism, bioinformatics, protein characterization, mode of action, functionality, digestibility and heat stability. Further information can be provided to support the weight-of-evidence should a hazard be identified in these studies. Characterization of identified hazards can be done through animal toxicity and dietary exposure assessment to address potential toxicity concerns.

Similarly, ERAs consider mode of action, activity spectrum, environmental exposure, and potential for hazard to NTOs, using a tiered testing approach to evaluate specific risk hypotheses. Food/feed assessments focus on human and animal health, whereas ERAs evaluate potential effects on non-target organisms, biodiversity, and other valued ecological entities. Consistent with safe-by-design principles, additional studies are conducted only when needed to address identified uncertainties and support overall safety conclusions. Notably, scientific publications may use different terminology or classification schemes for food and feed safety studies, including Tier I and Tier II studies [25] or core and supplemental studies [18]. Regardless of the terminology applied, these studies serve the same purpose: to support a problem formulation-based, hypothesis-driven, weight-of-evidence approach by addressing relevant questions identified during the assessment. For consistency, this review uses terminology aligned with current regulatory submission frameworks.

### 2.1. Safety Assessment of NEPs for Food and Feed

Food and feed safety assessments are conducted to evaluate the potential toxicity and allergenicity of NEPs in humans and animals. Consistent with Codex risk analysis principles, the safety assessment of NEPs follows a largely harmonized, structured, stepwise, weight-of-evidence approach on a case-by-case basis [10,25,26].

#### 2.1.1. Hazard Identification

This assessment focuses on identifying any potential hazards (toxicity and allergenicity) that may be associated with NEPs. Hazard identification of NEPs for food and feed safety integrates multiple lines of evidence to determine whether the protein is similar to proteins with HoSU or requires further evaluation.

##### HoSU

The protein, or a structurally and functionally related protein, is first assessed for a documented HoSU or history of prior consumption in food or feed. Additionally, the source organism of the introduced DNA is evaluated to determine whether it raises any toxicological or allergenic concerns. When the source organism has a well-documented HoSU, this information can provide relevant context and serve as a supporting line of evidence in the overall safety assessment of the NEP.

Importantly, while the source organism contributes to the weight-of-evidence, the safety assessment is focused on the NEP itself rather than the source organism. As the introduced gene typically encodes for only a single, defined protein, GM crops are characterized to confirm the absence of additional viable donor organism traits. Consequently, HoSU is evaluated on a case-by-case, weight-of-evidence basis. While a documented HoSU for the NEP or closely related proteins can provide supportive evidence for safety, the absence of such a history does not, by itself, indicate a safety concern. However, if the NEP is associated with potential safety concerns, additional studies may be warranted as part of a weight-of-evidence assessment. Instead, HoSU is considered alongside the other lines of evidence described below to identify and, where necessary, characterize potential hazards.

##### Bioinformatics Analysis

The amino acid sequence of a NEP is compared against databases of known toxins, allergens, and anti-nutritional factors. Lack of significant sequence similarity to entries in these databases, based on predefined and very conservative criteria, supports the conclusion that the protein is unlikely to exhibit toxic or allergenic properties. The toxin database consists of reviewed toxin entries in UniProtKB/Swiss-Prot, while the allergen database is derived from the HESI COMPARE database, a curated and publicly available resource of proteins with evidence of IgE binding used in regulatory safety assessments. Bioinformatic analyses are conducted using the following conservative screening criteria for potential concerns: no alignments exceeding the E-value threshold (e.g., ≤1 × 10^−5^) in similarity searches; no sequence identity greater than 35% over an 80-amino-acid sliding window, and/or no contiguous matches of 8 amino acids for allergenicity assessment [10,27,28].

In addition, bioinformatic analyses are also used to identify similarity to proteins with established HoSU or to commonly consumed dietary proteins. Such comparisons provide further context for evaluating familiarity and support weight-of-evidence considerations in protein safety assessment. Consistent with a safe-by-design approach, proteins with no biologically meaningful similarity to known toxins, allergens or anti-nutritional factors are typically prioritized for further development.

##### Protein Characterization and Equivalence Study

Characterization of NEPs produced in GM crops is a key element of safety assessment. Protein characterization integrates physicochemical, structural, and functional information to establish the identity, integrity, and biologically relevant properties of the NEP. Depending on the protein and its intended role in the assessment, relevant attributes may include amino acid sequence, molecular weight, post-translational modifications (e.g., glycosylation), immunoreactivity, biochemical properties, and biological activity. Together, these data support the characterization of the NEP and, where relevant, comparisons with related proteins, provide insight into the protein’s structure–function relationship and mode of action, and inform subsequent food and feed and ecological risk assessments.

Because safety studies often require quantities of purified protein that are difficult to obtain directly from plant tissue, particularly because expression levels in plant tissue are often low, test material is frequently produced in a heterologous expression system such as *Escherichia coli*. When such material is used, its suitability as a surrogate for the plant-produced NEP is demonstrated before use in safety studies. This equivalence assessment typically compares key physicochemical and functional characteristics of the plant- and heterologously produced proteins, including amino acid sequence, apparent molecular weight, immunoreactivity, glycosylation status (where relevant), and biological activity [27,28,29]. As part of this characterization, glycosylation status is evaluated to determine whether the plant-produced protein is glycosylated and to support the assessment of equivalence, rather than as a separate safety endpoint [10,18,29]. Where glycosylation differences are observed, their potential biological relevance, including potential effects on IgE binding and allergenicity, may also be considered [30,31,32]. Demonstrating that the heterologously produced protein is a suitable surrogate for the plant-produced NEP provides confidence that the resulting safety data are relevant and applicable to the overall risk assessment of NEP in the GM crop.

##### Mode of Action and Specificity

The biological function and mode of action (MOA) of the protein are well characterized as part of the safety assessment, with a defined and specific substrate or specific spectrum of activity. MOA refers to how a NEP produces its intended biological effect. Understanding the intended function (e.g., enzymatic activity, insecticidal specificity) supports a hypothesis-driven food and feed safety assessment by informing whether biologically plausible adverse effects could occur in humans or animals following dietary exposure. For insect control proteins, characterization of the MOA, target specificity, and biological relevance of the activity help determine whether biological activity is restricted to the target insect species and whether there is a plausible mechanism by which adverse effects could occur in humans or animals. Similarly, for enzymatic proteins, characterization of catalytic function, substrate specificity, and reaction products provides insight into potential interactions with endogenous biological pathways and the likelihood of off target effects. A well-characterized function and MOA provide confidence in the biological relevance and scientific basis of the safety assessment of the NEP.

##### In Vitro Digestibility and Heat Stability

The stability of NEPs is assessed under relevant conditions, including exposure to digestive enzymes and thermal processing. Rapid degradation or denaturation under predefined conditions provides information relevant to potential persistence in the gastrointestinal tract and systemic exposure.

In vitro protein digestibility (Figure 1) is typically evaluated using standardized enzymatic assays that simulate gastric conditions. Susceptibility to pepsin digestion is assessed using established, harmonized methods [33], with degradation monitored over time using appropriate analytical techniques [34].

Susceptibility to intestinal digestion (Figure 1) is further evaluated using pancreatin under conditions representing the upper small intestine. Protein degradation is assessed over time using appropriate analytical methods [34], providing complementary information to gastric digestion and supporting the overall assessment of protein stability.

To better approximate the digestive tract, sequential digestion assays are frequently conducted, in which the protein is initially subjected to pepsin digestion under acidic conditions, followed by pancreatin treatment at neutral pH [27]. In recent years, in vitro digestion has increasingly been considered more relevant to protein characterization, exposure assessment, and digested peptide assessment than to allergenicity prediction or hazard identification [20,35,36,37,38,39].

Heat stability (Figure 2) is evaluated by exposing a NEP to a range of temperatures (e.g., 25–95 °C) for defined time periods (typically 15–30 min). Following heat treatment, samples are analyzed for residual functional activity to determine the extent of thermal denaturation and loss of biological function [34]. Similar to in vitro digestibility, heat stability is increasingly considered more relevant to assessing potential exposure to biologically active protein following food and feed processing than to allergenicity prediction or hazard identification.

#### 2.1.2. Hazard Characterization

Hazard characterization further evaluates potential hazards identified during the hazard identification process of NEP assessment. When the weight-of-evidence assessment, including HoSU, bioinformatics analyses, MOA/functional specificity, and/or protein characterization, indicates a need for additional information, hypothesis-driven studies may be conducted on a case-by-case basis to address specific questions and refine the overall safety assessment, including animal studies.

Acute oral toxicity studies are conducted following accepted guidelines (e.g., EPA-OPPTS (870.1100)) designed to assess toxicity following a single exposure to a high concentration of a substance (i.e., the NEP). Animals are then monitored for morbidity, mortality, clinical observations, and changes in body weight for the subsequent 14 days at which point they undergo necropsy and evaluation of selected tissues. Initially, these NEP test substances were dosed based on estimated exposure levels with an additional safety factor applied. However, after the consistent lack of toxicology findings across NEP evaluations, these studies have shifted to utilizing limit doses. Although a limit dose of 2000 mg/kg body weight is considered appropriate under EPA-OPPTS 870.1100 guidelines, doses of up to 5000 mg/kg body weight have been utilized in response to requirements from some regulatory authorities. These limit doses are orders of magnitude greater than any potential exposure from food and feed.

Within the safety assessment framework, animal toxicity studies should be considered only when the weight-of-evidence assessment identifies a potential hazard requiring further evaluation. As proteins do not accumulate as a result of dietary exposure, an acute oral toxicity study is considered an appropriate model for evaluation of toxicity of a NEP in humans and other vertebrates when hazard characterization is scientifically justified. In certain cases, a 28-day repeated dose toxicity assessment may be requested for regulatory agencies as part of their standard data requirements following OECD guidelines [40].

Nevertheless, despite this intended role in hazard characterization, animal-based toxicity studies have been requested by regulatory authorities to provide further confirmation of NEP safety despite a lack of an identified and testable hazard hypothesis [21,28]. While an acute toxicity study can be appropriately utilized to characterize a hazard, its use for hazard identification in the absence of a testable hazard hypothesis is scientifically inappropriate [25].

#### 2.1.3. Exposure Assessments of the Newly Expressed Protein

Risk is assessed as a function of hazard and exposure. As such, human and animal exposure assessments are conducted to assess the anticipated exposure to NEPs through food and feed.

##### Animal Exposure Assessment

The dietary exposure of animals to NEPs in GM crops can be estimated using consumption values and body weight values to provide estimates of the maximum exposure that livestock animals can be expected to receive while still maintaining a nutritionally balanced diet.

Grain and silage used for animal feed would be expected to have gone through a series of blending steps with grain and silage from other sources prior to use in livestock and other animal feed. However, to evaluate animal exposure to NEPs in animal feed, 100% of the grain and forage utilized is assumed to be from the GM crop containing the NEP(s). This creates a very conservative “maximum possible” exposure scenario. Further intrinsic conservative assumptions are applied as the expression levels of the NEPs are used without adjusting estimated exposure or accounting for loss of the NEP as a part of grain or forage processing into meal or feed. Even when utilizing these highly conservative “maximum possible” exposure assumptions, anticipated animal exposure to NEPs from feed is extremely low, representing a negligible potential for risk.

##### Dietary Exposure Assessment

Either country specific consumption data or a global high exposed “eater-only” consumption values are used to estimate maximum realistic exposure of humans to the NEPs in a GM product as part of food consumption. Similar to the conservative estimates in animal exposure assessments, 100% of the foods derived from a crop are assumed to be from the GM grain containing the NEP(s). This is a conservative estimate as most GM crops entering the food supply and processing chain would be expected to have gone through a series of blending steps with other varieties prior to consumption. To further ensure that exposure estimates represent a maximum scenario rather than a realistic exposure level, the expression levels of the NEPs are used without consideration of any loss of protein resulting from food processing or cooking, as demonstrated in heat stability and digestibility studies. Even when utilizing these highly conservative “maximum possible” exposure assumptions, anticipated oral exposure of humans to NEPs is extremely low, representing a negligible potential for risk.

##### Dietary Risk Assessment

Dietary risk assessment, similar to dietary exposure assessment, can provide additional context for any potential hazards identified for NEPs. In the absence of adverse effects attributed to a NEP in acute toxicity studies, a dietary risk assessment is not scientifically necessary and an exposure estimation can be utilized as defined in section of Dietary Exposure Assessment. Should a dietary risk assessment be requested, human exposure estimates from consumption of GM products as food are compared to the no observed adverse effect level (NOAEL) derived from a toxicity study.

In addition to the assessments described above, other safety considerations may be addressed on a case-by-case basis [18,20]. Immunotoxicity testing is not generally a core component of NEP safety assessment but may be considered when the overall weight of evidence identifies a biologically plausible immune-related concern [17,18,25]. Longer-duration studies, such as 90-day rodent feeding studies, typically use whole GM grain or meal rather than the isolated NEP as the test substance. Such studies assess the safety of the GM food or feed as a whole and are therefore distinct from the NEP-focused safety assessment addressed in this review [17,18,41,42].

### 2.2. Ecological Risk Assessment of NEPs

Ecological risk assessments that assess for potential adverse effects on beneficial NTOs are required by countries when products expressing insect control proteins are intended for cultivation. An ERA for import approval is uncommon given that environmental exposure is low under import only conditions. Regulatory requirements for ERAs for insect control products demonstrate the value that societies place on ecosystem services, such as biocontrol, pollination, and decomposition, provided by beneficial NTOs and biodiversity both within agricultural fields and nearby areas. The basic framework of an ERA includes the problem formulation phase where risk hypotheses are developed based on protection goals; the analysis phase, where data are generated to address risk hypotheses identified during problem formulation; and risk characterization where information is integrated to determine risk under realistic conditions [11,12,43]. Ultimately, risk is characterized by combining information on both hazard (i.e., toxicity) and exposure.

#### 2.2.1. Increasing Importance of Problem Formulation for Newly Expressed Proteins

Problem formulation is a critical first step in the ERA process, but problem formulation is often revisited as new information is available [11,43,44]. The problem formulation stage begins early during product development where safe-by-design principles are applied (see more on safe-by-design below). A country’s protection goals, i.e., what aspects related to NTOs that a country wants to protect, are a key consideration during problem formulation and define the scope of an ERA [43,44]. Examples of common protection goals related to NTOs include protecting biodiversity and beneficial NTOs that provide ecosystem services. The more specific protection goals are, the more an ERA can be customized to address them [45,46]. Protection goals and other information gathered during problem formulation, such as HoSU, mode of action, and activity spectrum of the protein, are used to identify relevant risk hypotheses on potential pathways to ecological harm that can be further assessed using scientific rationale or study data. Information leveraged for problem formulation may already exist or may be developed specifically for a product. For example, some activity spectrum information may already exist in the literature for the protein of interest and related proteins; however, an initial activity spectrum study is often conducted covering a range of species during product development to help establish baseline knowledge about the protein. Ultimately, a more extensive understanding of the activity spectrum of an insecticidal protein is achieved by combining existing information, the initial activity spectrum screen, and the subsequent tiered approach beneficial NTO testing conducted in the analysis phase (see below).

Problem formulation has become increasingly important for the ERA over the last decade as novel insecticidal proteins have been developed with less familiarity compared to three-domain Cry proteins from *Bacillus thuringiensis* (Bt) with a long HoSU. Novel aspects of NEPs relevant to problem formulation include proteins derived from new source organisms or synthetic biology approaches, protein structural diversity, targeting new pests, new modes of action, increased activity spectra, products expressing multiple proteins, and proteins that are present at very low abundance or are difficult to isolate and characterize (e.g., membrane-associated or poorly soluble proteins).

#### 2.2.2. Tiered Non-Target Organism Testing

The analysis phase of the ERA includes NTO testing which is conducted using the tiered testing approach [12,14] (Figure 3). Though the term “tiered” is the same as mentioned above for the food and feed assessment and the concept is similar, the specifics of tiers in the case of the ERA differ. The tiered approach to NTO testing has been thoroughly discussed elsewhere as indicated by the references cited above; however, it is worth summarizing here to provide a structure to discuss relevant ERA-related trends and developments that have occurred over the past decade. For the tiered approach to NTO testing, Tier 1 consists of an initial hazard assessment where a set of representative surrogate NTO species are typically exposed to a high dose relative to a realistic environmental exposure of purified protein incorporated into an artificial diet. As discussed for food and feed safety studies, protein for NTO studies is produced via heterologous expression system since it is difficult to extract sufficient quantities of protein from the plant. Subsequent tiers are conducted if further characterization of the level of toxicity is required or to further clarify effects observed in early tiers under progressively more realistic conditions.

Surrogate species included in Tier 1 testing focus on selection of a limited number of typically beneficial taxa that represent ecosystem services [12,47,48]. The number of species needed for Tier 1 testing is not specifically prescribed but is informed by various factors such as published general guidance [14,48,49], leveraging guidelines from related products such as microbial pesticides (EPA Microbial Pesticide Test Guidelines, Series 885), historical precedent [50,51], and the case-by-case nature of each protein. In general, the more familiar a protein is, including a narrow activity spectrum, the fewer NTO species need to be tested; whereas more species are potentially required for a protein that is less familiar with a broader activity spectrum [49]. Familiar proteins with narrow activity spectra have often been utilized for GM crops over the last decade and the number of Tier 1 NTO species and other ERA studies has been proposed for further reduction by the U.S. EPA based on learnings from 30 years of conducting ERAs on these products [19]. Also relevant to this discussion are products with less familiar NEPs with broader activity spectra that in some cases have included additional Tier 1 NTO tests and higher tier testing. Broader activity spectra of some NEPs have often included effects detected in Tier 1 studies, prompting ERA questions that were infrequently encountered during the first twenty years of GM product development, such as what level of effect triggers higher tier testing.

USDA APHIS and EPA [14] and US EPA [51] indicate that a relevant effect in a Tier 1 test that would trigger further testing would be greater than 50% mortality at a dose 10 times higher than highest plant expression (e.g., 90th percentile of highest expressing tissue values). Likewise, if an effect is less than 50% mortality in a Tier 1 test, then further testing for that taxon is not necessary based on the assumption that the effect would no longer be present under realistic, lower exposure conditions and/or that some level of effect is acceptable. A Tier 2 test is designed to either further characterize sensitivity of a NTO (e.g., dose response) or assess the relevance of an effect detected in Tier 1 under more realistic but still highly controlled lab conditions such as exposing the NTO to plant tissue (e.g., pollen in the case of generalist predators that facultatively frequently feed on pollen) or lyophilized plant tissue incorporated into artificial diet. Higher tier testing (Tiers 3 and 4) is used to provide further context under still more realistic conditions. Tier 3 tests add realism while including some level of controlled conditions such as whole plant assessments in the greenhouse or screenhouse, or tritrophic studies, whereas Tier 4 studies include assessments under highly realistic conditions in an open field setting. The types of studies, study design, and specific triggers for Tier 3 and Tier 4 studies are more open to interpretation since it would be difficult to develop specific guidance on the varied options for higher tier refinement studies which are driven by the unique set of information for a given insecticidal protein (Figure 3).

#### 2.2.3. Assessing Proteins with Expanded Activity Spectra

As mentioned above, a discussion on higher tier test options and triggers has had increasing relevance over the past decade since some NEPs have had expanded activity spectra including new target pests and effects on NTOs both within the order of targeted pests and effects on insects in multiple orders (i.e., cross-order activity). Prior to the mid 2010’s, examples of lab level effects from commercialized insecticidal proteins on NTOs were sparse involving one example of a within order effect on an NTO and one example of cross-order activity involving an NTO [51,52]. Up to that point, ERA had reached conclusions that effects on beneficial NTOs in the field were unlikely for GM crops and those conclusions were based on results from Tier 1 and Tier 2. In those cases, use of lower Tier studies was appropriate since there were either lack of NTO effects or Tier 2 test results sufficiently indicated that NTO effects at the field level were not expected. More recently, however, additional examples of within-target pest order activity and cross-order activity on NTOs have occurred and some of those ERAs have included higher tier testing [50,53,54] (see Mpp75Aa1 ERA case study, Section 3.4.2). In each case, the existing ERA and NTO testing paradigm provided the framework to effectively assess the potential risk to NTOs of the NEPs with expanded activity spectra. The key difference in the ERAs of the more complex insecticidal traits relative to previous traits was that the novel proteins required additional consideration during problem formulation as well as utilization of higher tiers of the tiered testing approach that had not been necessary for previous proteins.

#### 2.2.4. From Hazard Identification to Ecological Relevance

The ERAs conducted for some NEPs over the last decade have further raised the question of whether there is an effect level in the field that would be acceptable for products considered reduced risk such as insect-resistant GM crops. It is expected that insect-resistant GM crops would minimally have some level of arthropod population reduction simply based on the intended reduction of target pests, which could reduce prey base for predatory and parasitic arthropods [51]. This has been documented for parasitic insects that specialize on the target pest [55,56] and also attributed to modest reductions in generalist predators as well [57]. Any potential reduction in beneficial NTO populations based on effects of an insect resistant protein characterized during tier testing would be assessed collectively within the totality of information generated for the ERA. For example, Blümel et al. [58] suggested that a 50% effect level of a non-target arthropod in the field could be an acceptable effect level since an arthropod population would presumably be able to recover from that reduction. Additionally, context on the relevance of an apparent population level effect on a generalist predator could be provided by considering the potential for compensatory biocontrol contributed by other generalist predators in the system. This functional redundancy has been demonstrated by modeling simulation and experimentally in Bt cotton fields [59,60]. Another concept for assessment of potentially acceptable effect levels is employed by the U.S. EPA where a conclusion of “no unreasonable adverse effect” must be reached for crop protection products [61]. This approach includes a risk-benefit analysis where the risk profile of a new product is compared to that of existing pest management practices and insecticide options to determine the acceptability of potential effects from the new product.

#### 2.2.5. Refining Margin of Exposure Approaches for NEPs

A key concept used in the ERA of insect-resistant GM crops is margin of exposure (MOE). MOE is simply hazard (toxicity) divided by exposure (such as NEP expression) and when both values are the same, the MOE = 1 and the hazard will be realized so long as the exposure value is realistic. Therefore, a higher MOE indicates a larger gap between the level of protein that has a toxic effect on an NTO and the level of protein that the NTO may actually be exposed to. Using different endpoints to represent hazard (e.g., effect concentrations vs. no effect concentrations) and different exposure scenarios (varying from highly conservative to realistic) will affect how an MOE will be interpreted. For example, if hazard is a lethal concentration that causes 50% mortality (LC_50_) of 10 µg protein/ml of diet and exposure is 10 µg protein/g pollen, then a population of lady beetles feeding on solely on pollen from the GM crop would be expected to experience 50% mortality. However, if hazard is represented as a no effect concentration (NOEC), then no meaningful effects would be expected to occur for the lady beetle population. In addition, since lady beetles frequently do not feed solely on pollen, a more realistic exposure value could be used that could result in a higher MOE for the example where an LC_50_ was used for hazard. So, MOE is an informative term, however the context of how it is used is important. From the early days of ERA for insect-resistant GM crops, the expectation for Tier 1 testing was that doses would be targeted at 10–100 times greater than plant expression. This is an effective starting point to inform a risk assessment since it is reasonable to assume that if an NTO is not sensitive at that high exposure, it is highly unlikely to be affected under realistic field conditions. A large MOE also serves as a safety factor for extrapolating findings from surrogate test species to other non-target organisms with similar ecological functions or taxonomic relatedness that were not directly tested.

The rationale to test at dose levels at least 10× of highest expressing tissue for Tier1 tests is a highly conservative starting point, however it should not be viewed as a strict threshold that must always be achieved. The utility of the approach, as mentioned above, is that if there are no effects at that highly conservative high dose, then a conclusion of minimal risk can be reached with a high degree of confidence. However, a MOE of 10 calculated using highest expressing tissue can lack relevance, especially beyond early Tier testing, since a MOE calculated using realistic exposure would be much higher. Furthermore, recommendations for MOEs of 10 or greater are generally based on acute Tier 1 test methods intended to reduce uncertainty through highly conservative exposure scenarios. However, higher-dose testing (e.g., 10× or above) for early tier testing may not always be achievable, due to methodological limitations, or necessary, for example, when there are low levels of uncertainty [51], such as when high levels of knowledge exist for familiar proteins. Under these scenarios, MOEs below 10 for early tier testing can often be considered protective (see the ERA case study for Cry1B.2 and Cry1B.3, Section 3.3.2). In addition, initial recommendations for MOEs of 10 or higher often assume acute test methods [14]; however, sub-chronic and chronic test methods are typically used for Tier 1 testing for insecticidal traits, which add confidence and reduce uncertainty in the risk assessments, further supporting the use of lower MOEs. For example, an MOE of ≥1× for a chronic study when the MOE is based on a NOEC is indicative of minimal risk by EPA [62]. Consistent with this perspective, the U.S. EPA and USDA consider ecological risk to occur when adverse effects are observed at realistic field exposure levels, when MOEs minimally exceed the expected environmental concentration (i.e., MOE > 1) [14]. Developing refined MOEs based on more realistic exposure scenarios has been discussed historically [63] and recently been utilized for NEPs. This approach can provide important context when bridging ERA data from previous products to other products that have higher expression or when relevant effects are detected in early tier testing [22,23] (see also the Cry1B ERA case study, Section 3.3.2). For example, an MOE of 10 calculated with the highest expressing tissue can be refined based on more realistic exposure if effects are detected that require higher tier testing (see Mpp75Aa1 ERA case study below, Section 3.4.2). When an effect requires higher tier testing, the mindset transitions to “what is the likelihood of that effect resulting in population level effects under realistic field conditions”. Since the initial MOE of 10 was based on an unrealistically high exposure scenario plus a 10× safety factor, a more realistic exposure is now needed to provide context for further risk assessment on potential effects under field conditions.

#### 2.2.6. Thirty Years of Experience: Opportunities to Leverage Scientific Rationale

During the evolution of the ERA of insect-resistant GM crops, certain studies were required by regulatory agencies or conducted by product developers based on uncertainty or out of an abundance of caution. Some of these studies, such as environmental fate studies, aquatic invertebrate studies, and proactive local non-target organism field studies have been reconsidered based on regulatory assessment experience with similar NEPs. It may be possible to generate specific risk hypotheses to address environmental exposure scenarios, or uniqueness of biodiversity within a given country; however, during problem formulation it may also be recognized that certain risk hypotheses can be addressed using scientific rationale instead of actual study data. This is exemplified by the recent guidance document published by U.S. EPA which recognizes increased opportunities to leverage scientific rationale based on 30 years of experience with ERA of insect resistant GM crops [19]. Importantly, the guidance document leaves open the opportunity to require study specific data in cases where problem formulation indicates sufficient uncertainty.

Some examples where scientific rationales and data reductions may be leveraged include when insecticidal proteins are similar to proteins produced in previous products (i.e., bridging from one product to another), where activity spectra of insecticidal traits are very narrow, or when a long history of data generation on a certain topic (e.g., low exposure to aquatic invertebrates) indicates that risk is unlikely.

Evaluation for the potential for interactions of insecticidal traits combined through breeding stacks or transformation vectors is another area where scientific rationale has been increasingly leveraged. Historically, interaction studies have been conducted to evaluate whether combined insecticidal traits produce synergistic effects. However, the accumulated evidence indicates that synergistic interactions among insecticidal proteins are uncommon and generally low in magnitude, and reviews of stacked Bt crops have not identified greater risks to non-target arthropods than those associated with the corresponding single trait crops [64,65]. Consistent with this evidence, the updated U.S. EPA guidance referenced above indicates that scientific rationale may be used in place of laboratory testing when no robust hypothesis exists for a synergistic interaction among combined PIPs [19]. Additionally, EFSA reviewed 61 applications for authorization of stacked events for food and feed safety submitted between 2003 to 2025, with the large majority of stacks conferring herbicide tolerance or insect resistance. None of the 61 stack applications evaluated by EFSA were indicative of an unexpected interaction [66].

#### 2.2.7. Regulatory Engagement and Adaptive Risk Assessment

As has been evident with some NEPs over the last decade, there will always be gray areas regarding whether a data requirement could be satisfied with scientific rationale or study data. Recognizing this, the U.S. EPA encourages product developers to have pre-consultations with the agency to share information on the product and the planned ERA approach [19]. These consultations provide regulatory agencies with the opportunity to become oriented to the new product and clarify data needs while allowing product developers to become aware of potential areas of concern and develop additional data if needed. Given the case-by-case nature of ERAs for GM crops, consultations with EPA and other regulatory agencies on topics such as the increased complexity of some NEPs have been helpful in ensuring that regulatory data needs are addressed (see Section 3.3.2, Section 3.4.2 and Section 3.5.2).

Adapting towards more fit for purpose ERA data based on many years of ERA experience has increased focus on opportunities for data transportability, i.e., using data previously generated in one country or region to support an ERA in another geography. This practice can avoid redundant generation of data and increases regulatory efficiency while maintaining scientific rigor [24,67]. One area where data transportability has particular utility is for NTO field studies that are commonly repeated across countries without serious problem formulation to determine whether a study is necessary. This is important since a key tenet of the tiered approach to NTO testing maintains that lower tier data should drive formulation of risk hypotheses for higher tier studies, such as field studies [12]. Since repetitive NTO studies conducted across countries are redundant and often not driven by lower tier results, leveraging existing NTO field data from other geographies should be considered. The transportability of NTO field data across geographies is supported by a growing body of literature [56,68,69]. Importantly, regulatory bodies are increasingly considering opportunities for data transportability and codifying the practice in updated regulations [70,71].

Other risk assessment topics or studies are sometimes placed under environmental or ecological risk assessment include horizontal gene transfer, weediness, gene flow, potential effects on soil microbes, and unintended effects. These topics are not discussed here since NEPs have generally not driven additional data collection or regulatory concerns in these areas. Increased use of scientific rationale and data transportability have often been leveraged to address questions or requirements for these topics since risk in these areas has not been identified over many years of risk assessment and cultivation of GM crops.

### 2.3. Safe-by-Design Considerations

The hazard identification and characterization framework for NEPs described above is complemented by a safe-by-design approach, whereby safety considerations are integrated early in the development and selection of NEPs. This includes preferential selection of proteins derived from source organisms with HoSU or established biological familiarity, lacking meaningful similarity to known toxins, allergens, or anti-nutritional factors, and exhibiting susceptibility to gastrointestinal digestion conditions. Biological specificity and mode of action are also considered to ensure that the NEP is both specific toward the intended target and is not plausibly associated with either toxicity or allergenicity in humans, livestock or non-target organisms. In addition, NEPs are often designed to optimize expression patterns, such as tissue specificity, expression level, developmental timing, and reduced expression in edible tissues (e.g., grain or seed), where feasible, to minimize potential unintended exposure. More broadly, protein design and engineering aim to avoid unintended changes in functional properties that could increase hazard potential. By aligning protein design and selection with established safety principles, safe-by-design considerations can streamline the overall regulatory safety assessments and support a more efficient weight-of-evidence evaluation, while reducing the likelihood that additional testing will be needed to address avoidable safety concerns. These considerations should complement rather than replace case-by-case safety assessment.

## 3. Case Studies of Newly Expressed Proteins

The following case studies illustrate how the principles described above including weight-of-evidence evaluation, bridging/read-across, and consideration of HoSU, structure/function relationships, and protein properties are applied in the safety assessment of NEPs. These examples highlight how different regulatory considerations are addressed for NEPs with varying levels of familiarity, structural complexity, and functional characteristics. The selected case studies concern insecticidal and herbicide-tolerance proteins and are intended to illustrate the application of these principles; they are not intended to encompass the full range of traits or potential NEP functions in GM crops. Additional reviews and examples involving other traits and NEP functions have been reported in the literature [27,41,72,73,74,75,76,77,78].

### 3.1. DMO

Dicamba monooxygenase (DMO) is presented here as an illustrative example of the transition from de novo safety assessment to the application of bridging and functional equivalence approaches for homologous proteins. This case highlights how cumulative safety data generated across multiple related variants can be leveraged to support subsequent assessments in a scientifically robust and efficient manner.

DMO proteins are members of the Rieske oxygenase family and catalyze the oxidative demethylation of dicamba, thereby conferring herbicide tolerance in GM crops [79]. Structural analyses have shown that DMO adopts a conserved homotrimeric architecture, with each monomer containing a Rieske [2Fe-2S] cluster and a non-heme iron catalytic center required for electron transfer and oxygen activation. The active site is specifically adapted for dicamba binding and catalyzes the exocyclic monooxygenation reaction underlying DMO function [79]. Early safety assessments of DMO-expressing crops were conducted on a case-by-case basis, with each variant evaluated independently using a standard weight-of-evidence framework, reflecting the limited prior experience and absence of accumulated safety data at the time. These evaluations incorporated multiple complementary lines of evidence, including HoSU considerations, bioinformatic analyses, physicochemical characterization, digestibility and heat stability assessments, and in vivo toxicological studies [21,80].

DMO is targeted to chloroplasts via a chloroplast transit peptide (CTP), enabling its proper localization and functional activity in planta [80]. Differential CTP processing and minor sequence optimization gives rise to multiple homologous variants, generally differing by small N-terminal sequence variations or limited amino acid substitutions, while maintaining high sequence identity (~90%) among variants (Figure 4A, Supplementary Table S1). Importantly, these sequence differences are primarily located outside functional domains [79]. Structural modeling demonstrated close alignment of multiple DMO variants with the crystal structure of the wild-type DMO protein (PDB ID: 3GTE), with root mean square deviation (RMSD) values < 1 Å and Template Modeling (TM) scores > 0.9 (Figure 4B, Supplementary Table S1), indicating strong conservation of three-dimensional structure. Consistent with this, biochemical analyses confirmed equivalent enzymatic activity, catalytic mechanism, and substrate specificity across variants, together with comparable physicochemical and immunological properties [21,80]. In addition, all variants exhibited similar stability profiles, being heat-labile and rapidly degraded under simulated gastric and intestinal conditions (Table 1).

Although no hazard was identified based on the core weight-of-evidence assessment, toxicological studies were conducted across multiple DMO variants, including 13 acute toxicity studies and five 28-day repeated-dose studies encompassing both first- and next-generation variants (e.g., MON 87708 DMO, MON 87419 DMO, MON 87429 DMO, KWS20-1 DMO and MON 94313 DMO) (Figure 4); no treatment-related adverse effects were observed. These studies, involving approximately 800 g of purified DMO proteins and over 1500 vertebrate animals, consistently demonstrated the absence of toxicological concern at high exposure levels [21,80]. These findings support bridging by demonstrating that, in the absence of identified hazards, toxicological studies consistently show no adverse effects and that minor sequence variations do not impact the safety profile of DMO proteins [21].

Overall, the convergence of structural, functional, and toxicological evidence across independently assessed DMO variants provides a strong experimental basis for applying bridging approaches. When structural and functional equivalence is established among homologous proteins derived from a common origin, safety data from a well-characterized variant can be extended to related variants, thereby reducing the need for redundant *de novo* studies.

This approach has been successfully applied in regulatory contexts, including submissions to the European Food Safety Authority (EFSA) for DMO-expressing crops, and is further supported by EFSA’s approval of MON 87419 maize, MON 94313 soybean and KWS20-1 sugar beet products, along with a growing body of peer-reviewed evidence [20,21,81]. Accordingly, the DMO case exemplifies how accumulated data and repeated weight-of-evidence assessments can enable a transition toward more efficient, science-based evaluation frameworks for homologous protein variants.

### 3.2. PPO

Protoporphyrinogen IX oxidase (PPO) proteins are presented here as an example illustrating the safety assessment of membrane-associated or intractable proteins, where conventional production and characterization approaches can be technically challenging. This case demonstrates how fit-for-purpose strategies, integrated within a weight-of-evidence framework, can support robust safety conclusions and regulatory approval while reducing data generation.

PPO is an enzyme involved in tetrapyrrole biosynthesis and is targeted to chloroplasts via a CTP, where it performs its biological function in planta [82]. Across GM crops, multiple homologous PPO variants have been generated through sequence optimization and differential CTP processing, resulting in minor N-terminal variations (Figure 5). The PPO expressed in GM crops (e.g., H_N90 PPO) is derived from *Enterobacter cloacae*, a bacterium widely distributed in soil, water, plants, and the gastrointestinal tracts of humans and animals. The H_N90 PPO utilizes a non-covalently bound flavin mononucleotide (FMN) cofactor for catalytic activity, a characteristic shared with other members of the HemG PPO enzyme family [83].

PPO belongs to a highly functionally conserved and essential enzyme class present across all domains of life. PPO enzymes including the HemY, HemJ, and HemG types are widely distributed across plants, including major food crops, animals, and microorganisms, with functional homologs identified in thousands of species, including beneficial and commonly encountered organisms such as probiotic bacteria [28,82]. Although these PPO classes exhibit substantial diversity in amino acid sequence and structural features, their catalytic function, the oxidation of protoporphyrinogen IX, remains strongly conserved across biological systems. This functional conservation is further supported by demonstrated interchangeability between PPO types (e.g., HemG and HemY) across species [82,84], supporting a shared biochemical role and evolutionary stability.

From both a functional and evolutionary perspective, PPO proteins lack characteristics associated with toxins or allergens and therefore do not suggest a biologically plausible hazard hypothesis for humans and animals. Consistent with this, the absence of hazard concerns from bioinformatic, structural and functional analyses, together with evolutionary functional conservation, indicates that toxicological studies may not be strictly required. Although no hazard was identified based on the core weight-of-evidence assessment, toxicological studies were conducted to satisfy regulatory requirements. Specifically, an acute oral toxicity study at doses up to 5000 mg/kg body weight and a repeated-dose toxicity study at doses up to 1000 mg/kg body weight/day were conducted. Consistent with Codex principles and the weight-of-evidence framework, a comprehensive safety assessment including bioinformatic analyses, structural and functional characterization, and toxicological assessments did not identify evidence of safety concerns (Table 2).

A key challenge in PPO assessment arises from its membrane-associated nature, which complicates recombinant expression, solubility, and purification, and limits the availability of native test material. To address these constraints, His-tagged PPO proteins were produced and used in safety studies. In addition, minor N-terminal sequence variations arise in planta from differential CTP processing, similar to observations with DMO. These retained N-terminal residues are short, flexible, and of plant origin, and do not exhibit sequence similarity to known toxins or allergens. Structural modeling further confirmed that PPO variants exhibit a high degree of structural conservation, with predicted structures showing high pLDDT (predicted local distance difference test) scores (Blue regions), indicating high confidence in the structural prediction and supporting strong structural similarity among the variants (Figure 5A). Experimental evidence demonstrates that neither His-tag incorporation nor CTP-derived N-terminal extensions affect PPO structural integrity, folding, enzymatic activity, or stability, and these findings are consistent with the structural modeling results, supporting the suitability of His-tagged PPO as a fit-for-purpose surrogate test material [28,85]. Therefore, large quantities of His-tagged PPO protein were produced and used as a surrogate test material for safety assessment of PPO proteins expressed in GM crops (Figure 5B).

The demonstrated structural and functional equivalence among PPO variants provides a strong scientific basis for applying bridging approaches to PPO safety assessment. Furthermore, the conserved biological function and widespread occurrence of PPO proteins support the absence of a plausible hazard hypothesis for food and feed safety. Under these conditions, safety data generated from a representative PPO protein can be scientifically extended to homologous variants, thereby reducing the need for redundant *de novo* studies. This bridging approach has been implemented in regulatory submissions for PPO-containing GM crops. By leveraging existing safety data from a representative PPO protein, redundant heat stability, in vitro digestibility, and toxicological studies were avoided, resulting in a reduction of approximately 800 mice used in toxicological testing while maintaining a robust, science-based safety assessment. This case illustrates how, even for technically challenging proteins, a scientifically robust safety assessment can be achieved through the integration of fit-for-purpose experimental design, weight-of-evidence evaluation, and bridging considerations. A summary of the safety assessments is provided in Table 2.

### 3.3. Cry1B.2 and Cry1B.3

#### 3.3.1. Food and Feed Safety Assessment

Cry1B.2 and Cry1B.3 are presented as examples illustrating the safety assessment of chimeric Bt Cry insecticidal proteins, in which functional domains are recombined from parental Cry proteins. This case highlights how domain-level conservation, functional equivalence, and weight-of-evidence evaluation can support robust safety conclusions while informing the appropriate use of bridging (read-across) approaches.

These proteins are derived from *Bacillus thuringiensis* (Bt) Cry proteins, a well-characterized class with an established HoSU. Cry1B.2 and Cry1B.3 were designed to retain the conserved three-domain Cry architecture (domains I–III), which mediates receptor interactions, membrane insertion and pore formation, while incorporating variations in the protoxin region [89]. Specifically, Cry1B.2 is comprised of domains I and II from Cry1Be, domain III from Cry1Ka2, and a protoxin domain from Cry1Ab (Figure 6). Cry1B.3 contains the same domains I–III as Cry1B.2, but differs in the protoxin domain, which is derived from Cry1Be. Importantly, prior studies have demonstrated that domain exchange strategies do not convert non-toxic protein domains into toxic entities from a mammalian safety perspective, nor do they alter the overall safety properties of the resulting chimeric proteins [90,91]. This principle provides a strong mechanistic basis for anticipating comparable safety profiles across engineered Cry variants.

From a sequence perspective, Cry1B.2 shares approximately 60% amino acid identity with previously safety-assessed Cry1 proteins (Supplementary Table S2), while its parental components Cry1Be and Cry1Ka have not been individually evaluated. In this context, the decision to conduct a de novo safety assessment of Cry1B.2 is scientifically justifiable, as it bridges between well-characterized proteins and less characterized parental sequences. Notably, the results from these Cry1B.2 studies, which comprise a full safety assessment dataset, including an acute oral toxicity study at doses up to 5000 mg/kg body weight and a repeated-dose toxicity study at doses up to 1000 mg/kg body weight/day, are consistent with the existing body of evidence on Cry proteins, showing no treatment-related adverse effects. These findings further support that minor sequence variation and domain recombination do not alter the established safety profile of Cry proteins when their structure, function, and mode of action are conserved (Table 2).

In contrast, Cry1B.3 shares 87% sequence identity with Cry1B.2 and retains identical functional domains (I–III) and the same mode of action. Structural comparison further demonstrated a high degree of similarity between the two proteins, with a low RMSD of 0.607 Å and a high TM-score of 0.862 (Figure 6 and Supplementary Table S2). Functional activity further revealed a high degree of similarity between the two proteins, with comparable activity against three target lepidopteran species: fall armyworm, European corn borer, and corn earworm [92]. Under a hypothesis-driven safety assessment paradigm, this high degree of structural and functional equivalence, combined with the absence of biologically relevant similarity to known toxins or allergens, does not support the need for additional de novo toxicological testing. Although no hazard was identified based on the core weight-of-evidence assessment, acute toxicity study at doses up to 5000 mg/kg body weight was conducted to satisfy regulatory requirements. As expected, the toxicological study for Cry1B.3 did not yield any contradictory or new safety conclusions beyond those already established for Cry1B.2 and related Cry proteins.

Collectively, these observations demonstrate that when domain architecture, mode of action, and safety-relevant characteristics are conserved, sequence variation within chimeric Cry proteins belonging to the same subfamily does not introduce new safety concerns. This case provides a clear example of how experimental data and accumulated knowledge can support the transition from de novo assessment to bridging for closely related protein variants, enabling efficient and scientifically justified safety evaluation without redundant testing.

#### 3.3.2. Ecological Risk Assessment

The ERA of Cry1B.3 provides an example of how a regulatory bridging approach can be used to leverage existing NTO safety data for one protein to effectively support the ERA of another similar protein and avoid unnecessary repetition of testing in the process. During problem formulation, it was determined that the ERA for Cry1B.3 could be bridged to the existing data for Cry1B.2 based on their shared mode of action, protein similarity, comparable exposure profiles, and the available hazard information for Cry1B.2. This bridging approach builds on previous regulatory precedents in which ERA data were bridged among products expressing the same protein (IPD072Aa) [22], between nearly identical proteins (Cry3Bb1 variants; 99.8% amino acid sequence identity) expressed in different maize products [93], and across different crop species expressing the same insecticidal proteins (Cry1A.105 and Cry2Ab2 in maize and soybean) [51,94]. The Cry1B.3 assessment represents a broader application of this paradigm because it bridges data between related, but less similar, proteins expressed in different crop systems; specially Cry1B.3 and Cry1B.2 share 86% amino acid sequence identity (Figure 6), and are expressed in different crop systems, cotton and soybean, respectively. Although Cry1B.3 and Cry1B.2 differ by 14% in amino acid sequence, the variation is limited to the C-terminal protoxin domain, which is not involved in receptor binding or insecticidal activity [95,96,97]. As a result, the proteins retain the same functional domains, mode of action, and target specificity, supporting the use of a bridging approach for ERA of Cry1B.3 to previously evaluated ERA of Cry1B.2 indicating negligible risk to NTOs [23]. Moreover, the activity spectrum of Cry1B.3 and Cry1B.2 is primarily limited to Lepidoptera [92] indicating minimal risk to beneficial NTOs, which further supported the bridging approach. Given the broader application of bridging to related proteins expressed in different crop systems, pre submission engagement with regulatory agencies provided an opportunity to discuss the scientific rationale for bridging approach, align on data requirements, and confirm that the proposed weight-of-evidence approach was appropriate for assessing Cry1B.3.

Based on this rationale, the Cry1B.3 ERA leveraged laboratory Tier 1 NTO studies previously conducted with Cry1B.2 [23]. These studies indicated no biologically relevant effects on NTOs from the Cry1B.2 protein under conservative laboratory conditions at concentrations exceeding expected environmental exposures [98]. Since there were no adverse effects at the high dose tested in Tier 1 NTO studies, no additional exposure refinement was necessary. Sufficient MOEs were determined for Cry1B.2 which remain protective of NTOs when accounting for differences in expression of Cry1B.3 compared to Cry1B.2 in the highest expressing plant tissues (Table 2) [23]. These MOEs were calculated using highly conservative exposure assumptions and no-observed-effect concentrations (NOECs) in Tier 1 NTO studies. Importantly, as discussed in Section 2.2.5, MOEs below 10 may still be considered protective when based on conservative exposure assumptions, limited uncertainty, and especially when based on a NOEC endpoint. For example, MOEs below 10 were considered protective for a bridging scenario involving Cry1Da_7 [23] due to the long history of safe use of Cry proteins, the limited activity spectrum of Cry1Da_7, and the predominantly chronic nature of the Tier 1 NTO test methods used to assess risk, for which NOEC-based MOEs ≥ 1 are considered protective by EPA [62]. Therefore, while achieving MOEs ≥10 based on highly conservative data can provide a helpful initial metric, the ability to leverage a bridging approach is ultimately best assessed based on the totality of available information. Additionally, refined MOEs can also be calculated based on more realistic exposure values to provide additional context [22,63].

Consistent with previous regulatory precedent, the combined evidence from protein relatedness, biological activity, conservative exposure assessment, and existing NTO hazard data supported a scientifically justified bridging approach, avoiding redundant NTO testing for Cry1B.3. This case study demonstrates that ERA bridging can be extended beyond near-identical proteins to more dissimilar, yet functionally equivalent, proteins expressed in different crop systems when sequence differences are confined to regions that do not contribute to insecticidal activity and the weight-of-evidence supports comparable hazard and exposure profiles. This case study further illustrates the value of regulatory engagement in facilitating scientifically justified bridging approaches, enabling targeted data reductions and ensuring fit-for-purpose ERA strategies for novel assessment scenarios. Overall, the Cry1B.2-to-Cry1B.3 bridging case illustrates how structure-function relationships, target specificity, exposure characterization, and existing NTO hazard data can be integrated during problem formulation to support an efficient and protective ERA, reducing redundant testing while maintaining confidence that Cry1B.3 poses negligible risk to beneficial NTOs and associated ecosystem services in agricultural systems.

### 3.4. Mpp75Aa1.1

#### 3.4.1. Food and Feed Safety Assessment

Mpp75Aa1.1 is presented as a case study for evaluating a member of the ETX/MTX2 family of β-pore-forming proteins (β-PFPs), illustrating how domain-level structural analysis, bioinformatic evaluation, and fit-for-purpose safety studies conducted within technical constraints can be integrated within a weight-of-evidence framework to support robust safety conclusions. Mpp75Aa1.1 is derived from *Brevibacillus laterosporus*, a microorganism with a documented HoSU [87]. As a member of the ETX/MTX2 family, it shares structural features with other proteins in this class, including certain mammalian toxins such as epsilon toxin and aerolysin, which provides a scientifically plausible basis for further evaluation within a safety assessment framework.

Proteins in the ETX/MTX2 family consist of three functional domains [99,100]. Domain I mediates receptor binding and toxin specificity and is structurally diverse, whereas domains II and III are responsible for pore formation and oligomerization and are structurally conserved across the family (Figure 7). Consistent with this architecture, structural superposition indicates close alignment of the conserved β-pore-forming core between Mpp75Aa1.1 and epsilon toxin, despite relatively low overall sequence identity (~26%), reflecting divergence outside conserved regions (Figure 7) [87,101]. Importantly, this conservation is not uniform across domains. The pore-formation and oligomerization domains show relatively higher structural similarity, whereas the receptor-binding domain exhibits substantially lower sequence (~19%) and structural similarity (Figure 7). Accordingly, residues associated with epsilon toxin receptor binding and mammalian toxicity are not conserved in Mpp75Aa1.1, and the receptor-binding domains cannot be structurally superimposed (Figure 7) [100,101]. Taken together, while overall structural similarity to epsilon toxin warrants further evaluation, such evaluation demonstrates that the domain-level differences, particularly in regions mediating receptor binding and target specificity, indicate functional divergence. Therefore, although Mpp75Aa1.1 shares limited similarity with epsilon toxin within conserved pore-forming domains, there is no scientific basis to support a plausible risk of toxicity to humans. Instead, this domain-level divergence supports functional differentiation, indicating that Mpp75Aa1.1 interacts with distinct, insect-specific receptors and lacks key determinants associated with mammalian toxicity. Accordingly, its mode of action is consistent with ETX/MTX2 family proteins active against insect targets and is not relevant to mammalian physiology. This case further illustrates that overall sequence or structural similarity alone is not sufficient to infer potential hazard. Rather, safety assessment should emphasize functionally relevant domains, particularly those mediating receptor binding and target specificity, highlighting the importance of domain-level criteria over global similarity when interpreting structural relatedness [100].

Consistent with proteins of low dietary hazard potential, Mpp75Aa1.1 is heat-labile (loss of activity at ≥55 °C) and rapidly degraded under simulated gastrointestinal conditions (proteolysis within <0.5 min), limiting the likelihood of systemic exposure to intact protein following consumption [87]. Toxicological evaluation further supports its safety profile. The maximum achievable dose levels in these studies were constrained by intrinsic physicochemical properties of the protein, particularly high viscosity at elevated concentrations, rather than limitations in formulation strategies. Despite extensive optimization of buffer composition, additives, and pH conditions, concentrations above approximately 30–35 mg/mL could not be achieved under physiologically relevant conditions without compromising protein integrity or introducing non-test-substance-related effects. Accordingly, the administered doses, approximately 2000 mg/kg body weight in the acute study and 600 mg/kg body weight/day in the 28-day study, represent maximum feasible exposure levels for hazard characterization. No treatment-related adverse effects were observed at these dose levels, which substantially exceed anticipated human and animal exposure to Mpp75Aa1.1 from consumption of MON 95275 grain [86,87].

Overall, the weight-of-evidence including HoSU, domain-level bioinformatic and structural evaluation emphasizing functionally relevant regions, rapid degradation under digestive conditions, lack of adverse effects at maximum feasible doses, and low anticipated dietary exposure supports the conclusion that Mpp75Aa1.1 can be assessed within an established safety assessment framework without concern for human or animal health. A summary of the safety assessment is provided in Table 2.

#### 3.4.2. Ecological Risk Assessment

The ERA of Mpp75Aa1.1 provides a useful case study for evaluating an insecticidal protein with biological activity extending beyond its primary target pest group. In addition to activity against corn rootworm target pests, Mpp75Aa1.1 showed activity in a limited number of lepidopteran pest species during activity-spectrum screening and activity in honeybee (*Apis mellifera*), green lacewing (*Chrysoperla rufilabris*), and ground beetle (*Poecilus cupreus*) under conservative Tier 1 laboratory testing conditions. The broader spectrum of activity identified through activity spectrum screening and Tier 1 studies was leveraged in problem formulation to help identify the taxa, exposure pathways, and risk hypotheses most relevant for subsequent evaluation. The ERA of Mpp75Aa1.1 also provides an example of assessing an insecticidal protein derived from a non-Bt source organism. Mpp75Aa1.1 originates from *Brevibacillus laterosporus*, a bacterium with documented HoSU but less regulatory familiarity in the context of insecticidal traits than traditional Bt-derived proteins. As such, the assessment illustrates how the established ERA framework applies equally well to NEPs regardless of the source of the insecticidal traits.

The progression beyond Tier 1 testing for Mpp75Aa1.1 was driven by the observed activity profile and resulting risk hypotheses rather than by the non-Bt origin of the protein itself. While Tier 1 studies are often sufficient to support ecological risk conclusions for insecticidal traits, the activity observed in beneficial arthropods from Mpp75Aa1.1 warranted additional refinement of both hazard and exposure. Consistent with the problem formulation process, the initial Tier 1 findings did not represent a conclusion of risk but rather informed refinement of the underlying risk hypotheses and identified areas of the ERA framework where additional data could improve understanding of the likelihood of adverse effects under realistic environmental conditions.

The evaluation of Mpp75Aa1.1 can be viewed alongside other published examples that reflect increasing diversity in both protein source organisms and biological activity profiles. For example, IPD072Aa from *Pseudomonas chlororaphis* exhibited activity against both corn rootworm and the beneficial convergent ladybird beetle (*Hippodamia convergens*), illustrating how activity outside the target pest group can require additional characterization within the ERA framework [22]. Similarly, IPD079Ea from the fern *Ophioglossum pendulum* represents a novel plant-derived protein developed for corn rootworm control [102], while IPD113 proteins identified from ferns, including *Pteris* species, demonstrate that novel insecticidal traits are increasingly being discovered outside traditional bacterial sources [103]. Together, these examples illustrate a broader shift toward proteins originating from increasingly diverse microbial and plant sources and encompassing greater structural and functional diversity than the early generations of insect-resistant traits.

Within this broader context, Mpp75Aa1.1 is particularly informative because it involved refinement through all four tiers of the ERA framework. Tier 1 studies were designed to conservatively characterize hazard across representative non-target taxa and functional groups using exposure concentrations substantially greater than expected environmental concentrations. Although effects were observed in lacewing, ground beetle, and honeybee larval assays, interpretation of these findings was informed by considerations of exposure realism, feeding ecology, and the likelihood of encountering equivalent concentrations under field conditions. Consistent with the problem formulation process, these findings did not indicate ecological risk but rather provided context to help guide higher tier studies and prompted additional consideration of more realistic exposure pathways.

A key component of the assessment was refinement of exposure estimates to better reflect how NTOs would encounter Mpp75Aa1.1 under field conditions. For honeybees, exposure was determined to be limited to pollen consumption, and expression of Mpp75Aa1.1 in pollen was negligible, resulting in a substantial MOE despite the effects observed under conservative Tier 1 laboratory testing. Given the lack of significant exposure, no additional testing with honeybees was required. For predatory arthropods, direct consumption of plant tissue represents an unrealistic exposure scenario, since predatory arthropods are primarily exposed through consumption of herbivorous prey that contains the protein in their tissue after feeding on the GM plant. To characterize this exposure route, concentrations of the Mpp75Aa1.1 protein were measured in a selection of representative prey that predatory arthropods would likely encounter in the agricultural ecosystem. These data provided a biologically relevant estimate of predator exposure through the primary route by which lacewings and ground beetles would encounter the protein in agricultural systems and the results of the assessment of protein levels in herbivorous prey demonstrated that prey-mediated exposure was considerably lower than protein levels in the plant and, as expected, far lower than the conservative assumptions used in the initial Tier 1 assessments.

The refined exposure characterization was integrated with subsequent Tier 2 concentration-response studies, a Tier 3 tritrophic study, and a Tier 4 multi-site field study evaluating carabid abundance and biological control function under representative agricultural conditions [50]. The incorporation of more realistic exposure estimates substantially reduced predicted exposure relative to conservative lower-tier assumptions and resulted in refined MOEs that supported a conclusion of minimal risk. The Tier-4 field study served as corroborative support under actual agricultural conditions despite the initial hazard response observed under worst-case laboratory exposures.

Collectively, this case study demonstrates that the current ERA paradigm—anchored in conservative screening, iterative exposure refinement, higher-tier ecological realism, and weight-of-evidence integration—is sufficiently robust to evaluate NEPs with broader biological activity spectra, more limited familiarity and HoSU, or novel sources and structural diversity than many historical commercialized insecticidal proteins (Table 2). As trait discovery increasingly expands beyond traditional *Bt* sources toward novel proteins from diverse bacterial and plant sources, the Mpp75Aa1.1 example provides evidence that the existing tiered ERA framework remains capable of resolving uncertainty and supporting scientifically defensible risk conclusions. This case study is also noteworthy because it illustrates how higher-tier testing can be tailored to address specific uncertainties identified during problem formulation. While general guidance exists regarding progression beyond Tier 1 when biologically relevant effects are observed, specific triggers and study designs for Tier 3 and Tier 4 assessments are often determined on a case-by-case basis. The Mpp75Aa1.1 assessment therefore provides a useful contemporary example of how iterative problem formulation, exposure refinement, and targeted higher-tier testing can be integrated to characterize risk for NEPs possessing broader activity spectra than many previously commercialized insecticidal traits.

### 3.5. Vip3Cb1

#### 3.5.1. Food and Feed Safety Assessment

Vip3Cb1 is presented here as an example illustrating the assessment of a member of the Vip3 family of insecticidal proteins, where structural familiarity with related proteins, bioinformatic evaluation, and standard hazard characterization are integrated within a weight-of-evidence framework to support robust safety conclusions.

Vip3Cb1 is derived from a *Paenibacillus popilliae* clade with limited documented HoSU according to regulatory review. It belongs to the Vip3 protein family, which is structurally distinct from three domain Cry proteins and exhibits a different domain architecture and activation process. However, Vip3 proteins undergo a conserved proteolytic activation process that results in the formation of a pore-shape tetramer and share an overall insecticidal mode of action similar to that of three-domain Cry proteins [104]. Bioinformatic analyses show no biologically relevant similarity to known toxins or allergens, indicating that unintended toxic or allergenic effects are unlikely [88].

Vip3Cb1 shares substantial sequence and structural similarity with other well-characterized Vip3 proteins, including 84% identity to Vip3Ca proteins and 66% identity to Vip3Aa proteins previously expressed in commercial GM crops. Structural analyses further demonstrate a high degree of similarity across the Vip3 family (e.g., RMSD < ~1 Å), with conserved domain architecture comprising N-terminal α-helical domains (DI–DII) and C-terminal β-sheet domains (DIII–DV) [104]. Functional characterization indicates a common mode of action involving proteolytic activation of the protoxin followed by formation of an active oligomeric complex, consistent with well-characterized Vip3 proteins [104]. Notably, the activated Vip3Cb1 quaternary structure is consistent with those reported for Vip3Aa16 and Vip3Bc1, including large conformational rearrangements in domains DI–DII that form an extended α-helical channel. This high degree of structural and functional conservation indicates that sequence differences between Vip3Cb1 and previously characterized Vip3 proteins do not alter key features relevant to biological activity or safety [88,104].

Vip3Cb1 is consistent with proteins of low dietary hazard potential. It is heat-labile, with loss of detectable activity after 15–30 min at temperatures ≥ 55 °C, and is rapidly degraded under simulated gastrointestinal conditions, with the full-length protein digested within approximately 0.5 min in pepsin [88]. These properties support a low likelihood of systemic exposure to intact, functional protein following consumption. Though there were not any hazards identified in the core weight-of-evidence assessment, acute oral toxicity study at doses up to 5000 mg/kg body weight performed to meet regulatory requirements did not identify any treatment-related adverse effects. Consistent with expectations, these findings align with the established safety profile of previously assessed Vip3 proteins (e.g., Vip3A).

Therefore, although Vip3Cb1 and its source organism do not have an established HoSU, the high degree of sequence identity (66%) and close structural similarity (RMSD < ~1 Å) [104] to well-characterized Vip3 proteins provide support for bridging within this protein family. Taken together, the weight-of-evidence including structural and functional conservation within the Vip3 family, absence of biologically relevant similarity to known toxins or allergens, rapid loss of activity with heat, rapid digestion, and safety conclusions from toxicology study, supports the conclusion that Vip3Cb1 can be assessed by bridging from related Vip3 proteins without concern for human or animal health [88]. A summary of the safety assessment is provided in Table 2.

#### 3.5.2. Ecological Risk Assessment

The ERA of Vip3Cb1 illustrates how accumulated experience from three decades of evaluating insect-resistant GM crops can be used to implement a fit-for-purpose risk assessment that focuses data generation on areas of uncertainty while leveraging existing scientific knowledge where risk hypotheses can be addressed through scientific rationale rather than additional testing [19,23]. During problem formulation, the insect-specific activity of Vip3 proteins, extensive familiarity with the Vip protein family, well-characterized exposure pathways, and previous regulatory experience with related proteins informed the assessment strategy and data requirements. Engagement with regulatory agencies further supported alignment on the risk hypotheses, data requirements, and opportunities to apply scientific rationale in place of additional testing. Consistent with a tiered ERA framework, conservative exposure estimates and Tier 1 NTO studies representing key ecological functional groups demonstrated no biologically relevant adverse effects and supported sufficient MOE, resulting in a conclusion of negligible ecological risk and no need for higher-tier testing (Table 2) [23].

A notable aspect of the Vip3Cb1 assessment was the application of data-reduction principles enabled by increased scientific familiarity, regulatory experience, and proactive regulatory engagement. Rather than generating data for all historically requested study types, several requirements were addressed through scientific rationale. For example, aquatic invertebrate toxicity, avian toxicity, and environmental fate or soil degradation studies were not conducted because existing knowledge indicated limited exposure, insect-specific biological activity, lack of biologically plausible hazard pathways to vertebrates, and rapid degradation of Vip proteins in the environment [19,41,105,106,107]. This approach is consistent with recent EPA guidance recognizing that certain studies may be unnecessary when problem formulation and existing evidence indicate low risk and limited uncertainty [19].

The Vip3Cb1 assessment therefore exemplifies the transition from a largely data-driven paradigm toward a more targeted, hypothesis-driven ERA that incorporates scientific rationale, protein familiarity, accumulated regulatory experience, and regulatory engagement. As reflected in recent EPA guidance, such fit-for-purpose approaches can reduce redundant testing while maintaining a protective assessment of ecological risk. More broadly, this case study demonstrates how knowledge of protein mode of action, activity spectrum, exposure pathways, environmental fate, and previous regulatory evaluations can be leveraged to focus data collection on decision-relevant uncertainties, improving the efficiency of future ERAs for related insecticidal proteins without compromising environmental protection.

## 4. Protein Processing and Sequence Variability: Relevance for Safety Assessment

Protein processing and sequence variability are common features of NEPs in GM crops and are key considerations in safety assessment frameworks. Variations such as minor sequence differences, domain recombination, or post-translational processing (e.g., truncation, cleavage, or N-terminal modifications) may arise from molecular design, expression systems, or natural biological mechanisms. These variations are evaluated within a weight-of-evidence framework, focusing on their impact on protein structure, function, and safety [20].

### 4.1. N-Terminal Processing

One important source of protein processing in GM crops is subcellular targeting, which is required to ensure proper localization and function. Nuclear-encoded proteins are translated in the cytosol, where N-terminal processing represents a fundamental and conserved step in protein maturation. In this compartment, removal of the initiator methionine is a ubiquitous co-translational event mediated by methionine aminopeptidases (MetAPs), which selectively cleave the N-terminal residue based on the identity and steric properties of the penultimate amino acid [108]. This process generates predictable Met-retained or Met-cleaved variants through proteolytic excision, without introducing additional covalent modifications beyond removal of the initiator residue [109,110]. Such N-terminal variability is widely observed across proteins in all organisms, including those expressed in GM crops, and reflects the inherent substrate specificity of endogenous MetAP enzymes rather than the genetic origin of the protein. Consistent with this, variation in initiator methionine retention has been reported for several transgenically expressed proteins (e.g., CspB, phosphinothricin N-acetyltransferase, Mpp51Aa2) without affecting biochemical activity, indicating that this processing is part of normal protein maturation [27,111,112].

For chloroplast-targeted proteins, additional processing occurs following subcellular localization. Many newly expressed proteins, including Cry1A.105, DMO, and PPO, are translated in the cytosol as precursor proteins with an N-terminal CTP that mediates import into the chloroplast [21,80,85,90]. Upon import into the stroma, the CTP is cleaved by stromal processing peptidase (SPP) and further degraded, enabling the protein to fold into its mature, functional form [113,114]. Depending on the N-terminal sequence context and the physicochemical environment within the chloroplast, additional N-terminal processing may occur, including alternative CTP cleavage and aminopeptidase-mediated trimming (e.g., methionine removal), resulting in minor heterogeneity at the mature N-terminus, as observed for MON 87708 soybean DMO (Figure 4). Such variability is commonly observed among chloroplast-localized proteins and represents an intrinsic feature of plant protein maturation. These processing events occur in multiple NEPs and are not expected to impact the protein function or safety profile of the mature protein.

### 4.2. Domain Exchange

In parallel to subcellular targeting, domain recombination (or domain exchange) is a well-established strategy used to optimize the activity of Bt Cry proteins. Cry proteins share a conserved three-domain structural architecture, enabling the recombination of domains from closely related proteins to generate chimeric variants with improved insecticidal spectrum or potency while preserving overall structure and mode of action [89,97]. Experimental evidence demonstrates that such modifications preserve functional equivalence without impacting mammalian safety [90,111]. For example, engineered Cry1A.105 as well as Cry1A.2 and Cry1B.2 were developed through recombination of domains from well-characterized Cry proteins, resulting in enhanced insect control while retaining the conserved mechanism of action and safety profile [89,90]. Similarly, the Cry1B.2/Cry1B.3 case study described herein demonstrated that exchange of the protoxin domain did not alter the safety conclusions for either protein. Importantly, domain recombination is not limited to engineered systems. Phylogenetic analyses indicate that domain exchange among Cry proteins also occurs naturally, contributing to the diversity of Cry proteins observed in *Bacillus thuringiensis* [115,116]. These findings demonstrate that domain recombination represents a predictable and biologically relevant source of variation that can be evaluated within established safety assessment frameworks.

### 4.3. Sequence Variation and Mutation

Beyond domain recombination, sequence diversification and mutation represent an additional, well-established source of functional variation in proteins used in GM crops. Targeted modifications through genetic engineering, as well as naturally occurring variation, can enhance protein potency or specificity without altering core structural and functional features. For example, engineered variants such as Mpp51Aa2 (formerly Cry51Aa2) and Cry1Da_7 demonstrate that increased insecticidal activity can be achieved through sequence optimization, including improved receptor interactions, increased target-insect potency, and, in some cases, efficacy against resistant insect populations [97,111,117]. Similarly, herbicide-tolerance proteins provide examples in which sequence variation or protein engineering can be introduced without altering the fundamental biochemical function or safety profile. For example, naturally occurring enzymes such as phosphinothricin acetyltransferase (PAT) and bialaphos resistance protein (BAR) exhibit sequence variation while maintaining equivalent biochemical function and safety profiles [118]. Likewise, the modified dioxygenase FT_T.1 was developed to enhance herbicide-tolerance performance relative to FT_T while retaining the same safety profile [119,120]. Importantly, these examples illustrate that sequence modification, whether engineered or natural, does not inherently alter the safety profile when the protein’s origin, mode of action, and functionally relevant domains are conserved. In parallel, modern protein design incorporates early safety considerations, where sequence changes are guided by preservation of structural integrity and avoidance of similarity to known toxins or allergens. This safe-by-design approach enables early de-risking during protein optimization and supports the conclusion that enhanced insecticidal potency can be achieved without introducing new safety concerns.

### 4.4. Affinity Tag

Additionally, the use of affinity tags (e.g., His-tags) represents a common, fit-for-purpose approach for producing and characterizing proteins used in safety assessment, when necessary. In cases where native protein production is technically challenging (e.g., low solubility or membrane association), His-tagged proteins can serve as suitable surrogate test materials [85,87,121,122]. Experimental evidence from the proteins evaluated demonstrates that the presence of a His-tag does not alter protein structure, enzymatic activity, or safety-relevant properties, supporting its use in toxicological and biochemical studies [28,87,121]. This approach enables robust data generation while ensuring that the test material remains representative of the native protein.

### 4.5. Protein Processing and Functional Activation

Protein processing is fundamental to the biological activity of many insecticidal proteins. Cry and Vip proteins are synthesized as protoxins and activated through proteolytic cleavage in the insect gut. For Cry proteins, this involves solubilization in the alkaline midgut followed by protease-mediated activation to generate the core toxin, which binds to specific receptors on midgut epithelial cells, leading to oligomerization, membrane insertion, and pore formation that disrupts cellular integrity and causes cell lysis [90,96]. Similarly, Vip3 proteins also retain highly conserved structural and functional characteristics despite sequence divergence, with variants such as Vip3Aa, Vip3Ca, and Vip3Cb1 exhibiting comparable folding, activation processes, and mode of action [88,104,123]. Accordingly, key structural and functional features are conserved within each subfamily despite sequence variability. The absence of biologically relevant similarity to known toxins or allergens within domains of concern further supports their safety. Collectively, this well-characterized mode of action depends on insect-specific gut conditions and receptor interactions that are absent in mammals or non-target organisms, thereby conferring target specificity without introducing new hazards.

Regulatory frameworks emphasize that safety assessment should focus on biological relevance rather than nominal sequence differences, integrating evidence on protein origin, function, exposure, digestibility, and toxicology [15,20]. This science-based approach avoids unnecessary duplication of studies while maintaining scientific rigor and aligning with the principles of the 3Rs.

In summary, these observations indicate that protein processing and sequence variability, arising from subcellular targeting, domain recombination, experimental design (e.g., affinity tagging), or biological activation, are expected and well understood. When such variations do not alter the mode of action or key functional properties, they do not introduce new safety concerns and can be addressed through bridging and established weight-of-evidence approaches, enabling efficient and scientifically robust safety assessments of NEPs in GM crops [21].

## 5. Bridging and Read-Across for Homologous Proteins

Bridging and read-across are related approaches used in the safety assessment of NEPs in GM crops. Read-across refers to the use of existing data from well-characterized, closely related reference proteins to inform the safety assessment of a target protein. Bridging refers to the supporting evidence demonstrating that the reference and target proteins are sufficiently similar in structure, function, and other relevant characteristics to justify the read-across. These approaches build on the principle that proteins sharing conserved structure, function, and mode of action are expected to exhibit comparable safety profiles, provided that any differences do not introduce biologically relevant similarity to known hazards (e.g., toxins or allergens) or alter domains relevant to hazard characteristics [20,21].

As discussed in the preceding sections, protein processing (e.g., chloroplast targeting and CTP cleavage), domain recombination, and sequence variability are common and well-understood features of proteins expressed in GM crops. Importantly, these processes generally preserve overall folding, domain architecture, and biological specificity, particularly within established protein families. When such key properties are maintained, proteins can be considered sufficiently similar to support the application of safety-relevant information across homologous variants.

This concept is supported by numerous examples across protein classes. For instance, domain recombination in Bt Cry proteins, enabled by their conserved modular architecture, has been shown to retain mode of action and preserve safety-relevant properties. Similarly, minor sequence variation or processing differences in enzymes (e.g., DMO, PPO) or insecticidal proteins (e.g., Vip3 family members) do not alter functional or safety-relevant characteristics when structural and functional integrity is preserved. Together, these observations provide a strong scientific foundation for applying bridging within and across homologous protein families.

Although the conceptual basis for bridging has been established, its practical application requires clear, operational criteria for determining similarity. Because no single universal threshold can define “sufficient similarity” across all proteins and assessment contexts, bridging or read-across should be implemented through a weight-of-evidence framework that integrates multiple complementary lines of evidence.

### 5.1. Bioinformatic Hazard Screening

Bioinformatic analyses are used to assess whether a NEP shares biologically relevant similarity with known toxins or allergens. This represents the most well-established component of the assessment, with applicable criteria described in Section of Bioinformatics Analysis. For bridging or read-across applications, bioinformatic analysis is a critical first step to determine whether a target protein introduces any new sequence similarity to known toxins or allergens relative to the reference protein. The absence of such similarities supports the application of existing safety conclusions to the target protein.

### 5.2. Sequence and Domain-Level Relatedness

Sequence similarity provides essential context for assessing relatedness within protein families, especially when interpreted alongside domain architecture and functional features. Established systems, such as Bt protein nomenclature, utilize sequence identity ranges (e.g., approximately 76–78%) to define subfamily groupings [124]. However, it is important to note that these values are not safety criteria and should not be treated as decision thresholds.

Empirical analyses indicate that proteins with relatively high sequence identity (e.g., over 70%) often share similar biological roles [125], and functional divergence tends to remain limited above approximately 60% identity [126]. In contrast, confidence in functional inference decreases progressively as sequence identity declines, with increased divergence more frequently observed below the ~50–60% threshold [126,127].

Consistent with these observations, sequence identity, when interpreted alongside domain architecture and conserved functional residues, can support bridging by indicating functional equivalence. In general, greater sequence conservation may increase confidence in inferred equivalence, whereas lower conservation typically increases uncertainty and warrants additional supporting evidence. Domain-level conservation adds further context, particularly for multidomain proteins, as conservation within functional domains has been associated with more reliable inference of protein function [128]. This underscores the importance of domain-based comparisons in conjunction with global sequence identity. Notably, these sequence similarity considerations do not align directly with bioinformatic screening criteria for toxins and allergens, as the objectives differ. Allergen and toxin bioinformatics are designed to maximize sensitivity and avoid missing any potential matches, whereas sequence-based comparisons for bridging aim to establish a high likelihood of functional similarity. These approaches are therefore complementary rather than contradictory, reflecting their distinct purposes.

Overall, sequence identity should be viewed as supportive rather than definitive evidence. Its interpretation must be integrated with considerations of domain architecture, active site conservation, and, where available, structural and functional data. Conclusions should explicitly acknowledge uncertainty and be made on a case-by-case basis within a weight-of-evidence framework [10].

### 5.3. Structural Conservation

Protein structure is a primary determinant of function, reflecting the three-dimensional arrangement of domains and other functionally important elements. Because protein structure is often more conserved than primary sequence, structural analysis can provide complementary evidence of relatedness, particularly when sequence identity is moderate or low. Backbone structural similarity is commonly evaluated using metrics such as TM-score [129] and RMSD [130]. TM-score assesses the overall similarity of protein folds and is less sensitive to local structural deviations, whereas RMSD quantifies the average distance between corresponding atoms after structural alignment and is more sensitive to local differences. A key feature of modern structure prediction tools is the inclusion of confidence metrics that enable rigorous evaluation of model quality. pLDDT provides a per-residue confidence score that reflects the reliability of the predicted local geometry, with higher scores indicating greater structural prediction accuracy [131,132,133,134,135]. Although the pLDDT score is optimized for backbone residues, side-chain positions are reasonably well predicted when the backbone pLDDT score is very high (>90) [136]. In addition to backbone and local folds, electrostatic potential mapping at relevant pH can evaluate the spatial distribution of surface charges of binding sites or exposed catalytic pockets [137]. Together, these in silico predictions distinguish protein shape and physicochemical similarity and can be correlated with similar function and molecular interaction behaviors [138].

The utility of these structural metrics is illustrated by homologous protein variants such as DMO (Figure 4B, Supplementary Table S1) and PPO (Figure 5A), which exhibit high in silico structural similarity across variants, as demonstrated by low RMSD values (<1 Å), high TM-scores (>0.9), and/or high-confidence structural predictions (pLDDT). Similarly, despite variation in amino acid sequence, Cry1 proteins retain a highly conserved three-domain architecture, and low RMSD and high TM-score values among Cry1 proteins evaluated further demonstrate conservation of their overall structural fold (Supplementary Table S2 and Figure S1). These examples highlight that predicted structural conservation can extend beyond primary sequence conservation and provide important evidence of functional relatedness. Notably, RMSD values may vary depending on the alignment algorithm and fitting procedure used. For example, methods that exclude poorly aligned regions may yield lower RMSD values than methods based on all aligned residues. Therefore, RMSD values should be interpreted within the context of the calculation methodology; however, the overall conclusion of high structural similarity among these protein variants remains unchanged.

Consistent with Section 5.2, predicted in silico structural similarity should be interpreted as supportive rather than definitive. High conservation, especially within catalytic or binding domains and at functionally critical residues, strengthens inference of functional equivalence and supports bridging or read-across. In contrast, structural differences, including changes in catalytic or binding domains or local variations (e.g., loops or surface features), may indicate altered specificity or function and increase uncertainty, as illustrated by differences in the receptor-binding domain and key receptor-binding residues between Mpp75Aa1.1 and epsilon toxin (Figure 7), which result in distinct receptor interactions and biological activities.

Domain-level structural conservation provides additional resolution, particularly for multidomain proteins. Preservation of domain folds and spatial configuration, even with moderate sequence divergence, is often associated with retained biochemical function, whereas domain loss, gain, or rearrangement may indicate functional diversification and warrants cautious interpretation. Where available, experimentally determined structures provide the highest confidence; however, high-quality computational models from in silico protein folding predictions can also be informative when supported by appropriate confidence metrics. Accordingly, protein similarity should be evaluated using a weight-of-evidence approach that integrates amino acid sequence identity with structural similarity metrics, including RMSD, TM-score, pLDDT scores, and domain-level conservation, rather than relying on any single parameter in isolation. Interpretation should consider model quality, structural coverage, and methodological limitations, and conclusions should be drawn on a case-by-case basis with uncertainty explicitly acknowledged [10].

### 5.4. Functional Equivalence

Functional assessment represents a critical determinant of bridging applicability. For enzymes, this assessment encompasses substrate specificity and catalytic activity; for insecticidal proteins, it includes conserved modes of action, activation processes, and biological specificity and activity. Demonstrating functional equivalence is essential to ensure that any sequence and structural differences do not lead to variations in hazard potential.

Functional equivalence can be evaluated using a diversity of statistical approaches. Equivalence testing is particularly valuable for demonstrating the absence of a meaningful difference. In this context, prediction interval (PI) based approaches are commonly applied to define acceptance limits for functional equivalence, where target protein(s) are considered functionally equivalent if their activities fall within the 95% PI derived from a reference protein [21,139,140]. In addition, hypothesis-testing approaches, such as *t*-tests, may be used to compare functional activity between reference and target proteins; however, these tests primarily assess statistical differences and should be interpreted alongside their biological relevance.

### 5.5. Phylogenetic Context

Proteins considered in this context are typically derived from a common evolutionary origin and belong to established protein families. Phylogenetic analyses help define their evolutionary relationships and provide context for interpreting observed sequence variation. This framework can help distinguish expected within-family variability from sequence changes that may warrant further evaluation for potential functional or safety relevance. For homologous protein variants derived from the same gene, such as those resulting from limited amino acid substitutions, insertions, deletions, or differential N-terminal processing (e.g., DMO and PPO variants), phylogenetic context can provide support for bridging. These variants generally represent different forms of the same protein, and minor sequence differences are not expected to alter the mode of action, biological function, or safety profile when functionally relevant domains are conserved. Consequently, an existing safety package may be leveraged when equivalence is scientifically demonstrated.

In addition, phylogenetic clustering can help identify closely related reference proteins and support selection of appropriate comparators for read-across and bridging. However, greater caution may be warranted when considering bridging between proteins derived from different genes. In such cases, phylogenetic relatedness, together with structural, biochemical, functional, and exposure data, may be needed to determine whether reliance on an existing safety package is scientifically justified. Agreement between phylogenetic relationships and structural or functional characteristics can further strengthen confidence in inferred similarity.

Bridging is supported by multiple complementary lines of evidence, each contributing to the overall weight-of-evidence assessment. Bioinformatic analyses help exclude biologically relevant similarity to known toxins and allergens; sequence identity and phylogenetic relationships establish relatedness among protein variants; structural analyses, including predicted or experimentally derived protein structures, assess conservation of three-dimensional architecture; and functional characterization confirms biological equivalence and relevance. When these lines of evidence collectively support similarity between proteins, safety data generated from a well-characterized reference protein can be extended to related proteins, reducing the need for redundant testing while maintaining scientific rigor. Bridging has been applied across diverse protein classes, including DMO [21,81], PPO [85] and Cry1B.2 and Cry1B.3 [23], where structural and functional conservation supports evaluation of homologous proteins. Within this framework, HoSU provides relevant contextual evidence, particularly when direct experimental data are limited.

## 6. Integration of Human and Ecological Risk Assessment and Global Regulatory Experience

The safety assessment of NEPs in GM crops has traditionally been conducted through complementary evaluations of food and feed safety and ERA. Although these assessment domains operate within distinct regulatory contexts, they are grounded in common scientific principles, including hazard identification, exposure assessment, and weight-of-evidence evaluation, applied on a case-by-case basis. Overall, these shared principles support a coherent and scientifically robust framework for assessing NEP safety across human, animal, and environmental protection goals.

### 6.1. Convergence of Core Scientific Principles

Across both food and feed safety assessments and ERA, a common conceptual framework supports regulatory decision-making. Although specific data requirements may vary among regulatory authorities, the underlying scientific principles have become increasingly aligned over time. Central to this framework is the characterization of protein identity, structure, function, and mode of action, together with bioinformatic evaluation of similarity to known toxins or allergens and consideration of likely exposure under conservative yet biologically relevant scenarios. Collectively, these elements provide the scientific foundation for a hypothesis-driven, tiered approach in which additional data are generated only when a plausible hazard has been identified. They also support the application of safe-by-design approaches, whereby knowledge of protein properties and exposure is incorporated early in the design and selection of target proteins to minimize potential hazards.

Although the specific risk assessment goals and assessment endpoints differ between contexts, the underlying scientific logic is consistent. Food and feed safety assessments focus on dietary exposure, digestibility, and the potential for toxicity or allergenicity, whereas ERA focuses on potential effects on NTOs and ecological functions. In both cases, however, risk characterization is based on the relationship between hazard and exposure. Conservative assumptions are typically used in early tiers to ensure protective outcomes, with subsequent refinement applied as needed to improve biological relevance and reduce uncertainty.

### 6.2. Integration of Weight-of-Evidence Across Assessment Domains

The case studies discussed in this review demonstrate that many of the core data elements used in NEP safety assessment are informative across both human health and ecological contexts. In particular, mode of action and biological specificity provide a common basis for evaluating the likelihood of unintended effects in mammals and non-target organisms. Likewise, protein stability contributes to exposure assessment in both contexts, with digestibility and heat stability informing potential dietary exposure to intact protein, and environmental degradation characteristics informing potential environmental persistence and exposure potential. Expression levels in plant tissues similarly inform estimates of dietary intake as well as environmental exposure for relevant ecological receptors.

Integration of these data within a unified weight-of-evidence framework supports consistent interpretation across assessment domains and can reduce unnecessary duplication of studies. This integrated approach also enables safe-by-design strategies, in which intrinsic protein characteristics and expected exposure profiles are considered proactively to guide protein selection and development. Furthermore, this convergence reflects the continued evolution toward protein-based assessment paradigms, in which intrinsic protein properties, biological specificity, and realistic exposure considerations are central to risk characterization.

### 6.3. Role of Tiered Testing and Exposure-Driven Refinement

Both food and feed safety assessment and ERA are implemented through structured approaches that integrate hazard identification, hazard characterization, exposure assessment, and risk characterization. In ERA, Tier 1 laboratory studies are intentionally designed to represent conservative exposure scenarios, thereby enabling sensitive detection of potential hazards across representative taxa. When effects are observed or uncertainty remains, higher-tier studies may be used to refine hazard characterization and exposure assessment under more realistic ecological conditions. Similarly, in food and feed safety assessment, hazard identification and characterization are informed by a weight-of-evidence evaluation of multiple complementary lines of evidence, while additional studies may be considered where scientifically justified.

Across the case studies examined in this review, the assessment framework consistently shows that initial conservative assumptions frequently overestimate exposure, whereas subsequent refinements improve biological realism and reduce uncertainty in risk characterization. Where no hazard has been identified, the overall evidence across both domains supports conclusions of negligible risk under realistic conditions of use and exposure.

### 6.4. Leveraging Global Regulatory Experience

Three decades of regulatory experience have generated an extensive body of knowledge across multiple frameworks, covering a broad range of proteins and trait categories. This collective experience provides strong empirical support for the robustness and reliability of approaches currently applied in regulatory submissions and approvals.

Across numerous case studies and regulatory evaluations, scientific conclusions have been highly consistent. These include the absence of biologically relevant toxicity or allergenicity for NEPs assessed under established food and feed safety frameworks, as well as the absence of adverse ecological effects on non-target organisms under environmentally relevant exposure conditions. Importantly, these conclusions have been consistent across proteins that differ in familiarity, including proteins belonging to well-characterized families and proteins with more limited histories of prior assessment.

This accumulated regulatory knowledge and experience has also informed the evolution of assessment practices. In particular, it has supported greater acceptance of bridging and read-across approaches, reduced reliance on routine animal testing in the absence of identified hazard, and increased emphasis on biological relevance and exposure-driven risk assessment.

Overall, the convergence of scientific principles across food and feed safety assessment and ERA, along with accumulated regulatory experience, provides a strong basis for more harmonized and efficient assessment approaches. Leveraging shared data elements within a common weight-of-evidence framework can improve efficiency while maintaining scientific rigor and protective standards. Consistent with these principles, a unified framework anchored in protein structure, function, biological specificity, and exposure therefore provides a reliable basis for assessing increasingly diverse proteins introduced through modern biotechnology.

## 7. Future Directions

The case studies presented in this review illustrate how many of these emerging approaches are already being applied in practice. DMO, PPO, Cry1B.2/Cry1B.3, and Vip3Cb1 demonstrate the increasing use of bridging strategies supported by complementary lines of evidence, including bioinformatics, structural and functional characterization, and HoSU, to extend existing safety knowledge to related protein variants. In contrast, Mpp75Aa1.1 illustrates how established risk assessment frameworks can accommodate proteins with reduced familiarity or broader biological activity while maintaining a hypothesis-driven, weight-of-evidence approach. Together, these examples demonstrate that existing safety assessment frameworks are sufficiently robust to evaluate increasingly diverse proteins. Advances in scientific understanding, familiarity, and regulatory experience can further support more targeted, fit-for-purpose assessments. Building on these experiences, several opportunities exist to further enhance efficiency, transparency, and scientific basis of future safety assessments.

### 7.1. Food and Feed Safety

#### 7.1.1. Bridging and Read-Across Approaches

A key future opportunity is the broader use of bridging and read-across approaches, where safety conclusions for NEPs are informed by existing data from structurally and functionally related proteins. Such approaches could be supported by transparent decision frameworks integrating HoSU, bioinformatics, sequence and domain conservation, structural similarity, functional equivalence, mode of action and exposure. Proteins demonstrating strong similarity across these categories may be evaluated using existing safety data from related proteins, while only proteins presenting uncertainty would require additional targeted studies.

#### 7.1.2. Exposure-Informed and Hypothesis-Driven Assessment

This direction, given the examples provided, aligns closely with recent EFSA initiatives, which emphasize HoSU, protein characterization, structural analyses, read-across, exposure assessment, and hypothesis-driven testing with biological context while reducing reliance on routine animal studies. Recent EFSA case studies demonstrate how mode of action, structural similarity, and clinical relevance can support robust safety conclusions without routine vertebrate animal testing [20].

Building on existing hypothesis-driven risk assessment frameworks, future assessments are expected to place greater emphasis on exposure-informed decision making, integrating protein expression levels, environmental fate, dietary exposure estimates, and biologically relevant exposure scenarios into the overall weight-of-evidence assessment. Such approaches would help ensure that data requirements remain proportionate to potential risk while maintaining a high level of safety protection.

#### 7.1.3. New Approach Methodologies (NAMs) and AI-Enabled Tools

Future assessments are also expected to increasingly incorporate NAMs, including advanced bioinformatics, protein structure prediction, protein language models, mechanistic in vitro assays, and systems biology approaches. These approaches are best viewed as complementary tools that can strengthen existing weight-of-evidence assessments rather than replacing established risk assessment principles. These tools can provide more informative and mechanistically relevant evidence while supporting the 3Rs principles of reducing, refining, and replacing animal testing. From a developer’s perspective, validated mechanistic NAMs could be valuable when integrated into established weight-of-evidence frameworks and linked to specific risk assessment questions. As these technologies continue to mature, they may enhance confidence in bridging, read-across, and other alternative approaches by providing biologically relevant evidence to address specific uncertainties.

#### 7.1.4. Reference Databases and Computational Resources

Another critical need is the development of curated reference databases containing well-characterized safe proteins, allergens, toxins, and protein families, including allergen ranking and clinical relevance [141]. Combined with advances in artificial intelligence and structural biology, these resources would support more consistent computational screening, facilitate bridging decisions, and improve transparency across regulatory evaluations.

### 7.2. Forward Looking Ecological Risk Assessment

Future ERAs are expected to build upon the established problem formulation and tiered testing framework while becoming increasingly fit-for-purpose. Three decades of experience with insect-resistant GM crops, together with recent regulatory guidance [19], support a greater emphasis on targeted data generation focused on key uncertainties identified during problem formulation. Future ERAs will likely make greater use of protein-specific information, including mode of action, receptor biology, activity spectrum, protein similarity, and ecological exposure pathways, to develop and test biologically plausible risk hypotheses. Expanding familiarity with insecticidal protein families may also enable broader application of bridging approaches, allowing existing hazard and exposure data to inform assessments of related proteins, new products expressing the same protein, or proteins with comparable biological properties when supported by a robust weight-of-evidence. Importantly, recent assessments of NEPs with broader activity spectra have demonstrated that the current ERA framework remains sufficiently flexible and protective, with higher-tier testing available when warranted. As scientific understanding and regulatory experience continue to grow, future ERAs are expected to rely increasingly on scientific rationale, bridging, and targeted testing, reducing redundant studies while maintaining robust environmental protection.

### 7.3. International Harmonization

Finally, although substantial convergence in scientific principles has already been achieved, greater international harmonization in regulatory implementation, data requirements, and acceptance of emerging methodologies will further improve efficiency and consistency. Alignment on concepts such as HoSU, bridging, read-across, protein similarity criteria, and NAM integration would reduce redundant testing, improve regulatory predictability, and support efficient innovation while maintaining a high standard of protection for human health, animal health, and the environment. Ultimately, the future of NEP assessment will likely be characterized by integrated, protein-based, hypothesis-driven frameworks that build upon established risk assessment principles while leveraging advances in biology, computation, and regulatory science to deliver more proportionate, efficient, and scientifically robust evaluations.

## 8. Conclusions

Over the past three decades, the safety and ecological assessment of NEPs in GM crops has evolved into a robust, science-based framework characterized by substantial convergence in the scientific principles reflected across regulatory frameworks worldwide. Grounded in Codex principles and implemented through a case-by-case, weight-of-evidence approach, this framework has demonstrated both protectiveness and adaptability across proteins with differing degrees of familiarity, structural complexity, and functional diversity.

The case studies presented in this review show that the core elements of NEP safety assessment, including hazard identification based on HoSU, bioinformatic, structural, and functional lines of evidence; exposure assessment; and hypothesis-driven characterization, where warranted, have supported scientifically robust safety conclusions for the NEPs evaluated to date. Importantly, these elements also enable the application of safe-by-design principles, whereby knowledge of protein structure, function, and exposure is incorporated early in the design and selection of candidate proteins to minimize potential hazards. For proteins with established familiarity or conserved biological function, accumulated knowledge can be effectively leveraged through bridging and read-across approaches. Proteins with more limited familiarity, however, can be assessed within the same structured weight-of-evidence framework.

A central theme emerging from these evaluations is the importance of biological relevance. Protein properties, including mode of action, structural and functional conservation, digestibility, and exposure, provide a sound scientific basis for assessing hazard potential across human health and ecological endpoints. When considered early in the development process, these properties can inform safe-by-design strategies that reduce the likelihood of adverse outcomes. Where these lines of evidence do not support a plausible hazard hypothesis, additional testing, including routine animal studies, is unlikely to contribute meaningfully to safety conclusions, consistent with the application of 3Rs principles.

The integration of food and feed safety assessment with ERA further underscores the convergence of scientific principles across regulatory contexts. Shared reliance on protein characterization, exposure assessment, and weight-of-evidence evaluation supports a coherent and efficient assessment paradigm that spans human, animal, and environmental contexts.

Overall, the evidence reviewed here indicates that current NEP safety assessment frameworks are fit for purpose and adaptable to emerging challenges. Continued application of weight-of-evidence, hypothesis-driven approaches, together with proactive integration of safe-by-design considerations, advances in scientific understanding, computational tools, and further harmonization of regulatory implementation, including consideration of the Cartagena Protocol on Biosafety [142] and its provisions for risk assessment, transboundary movement, and post-release monitoring and surveillance, will support the efficient, proportionate, and scientifically rigorous assessment of increasingly diverse proteins in agricultural biotechnology.

## Figures and Tables

**Figure 1:**
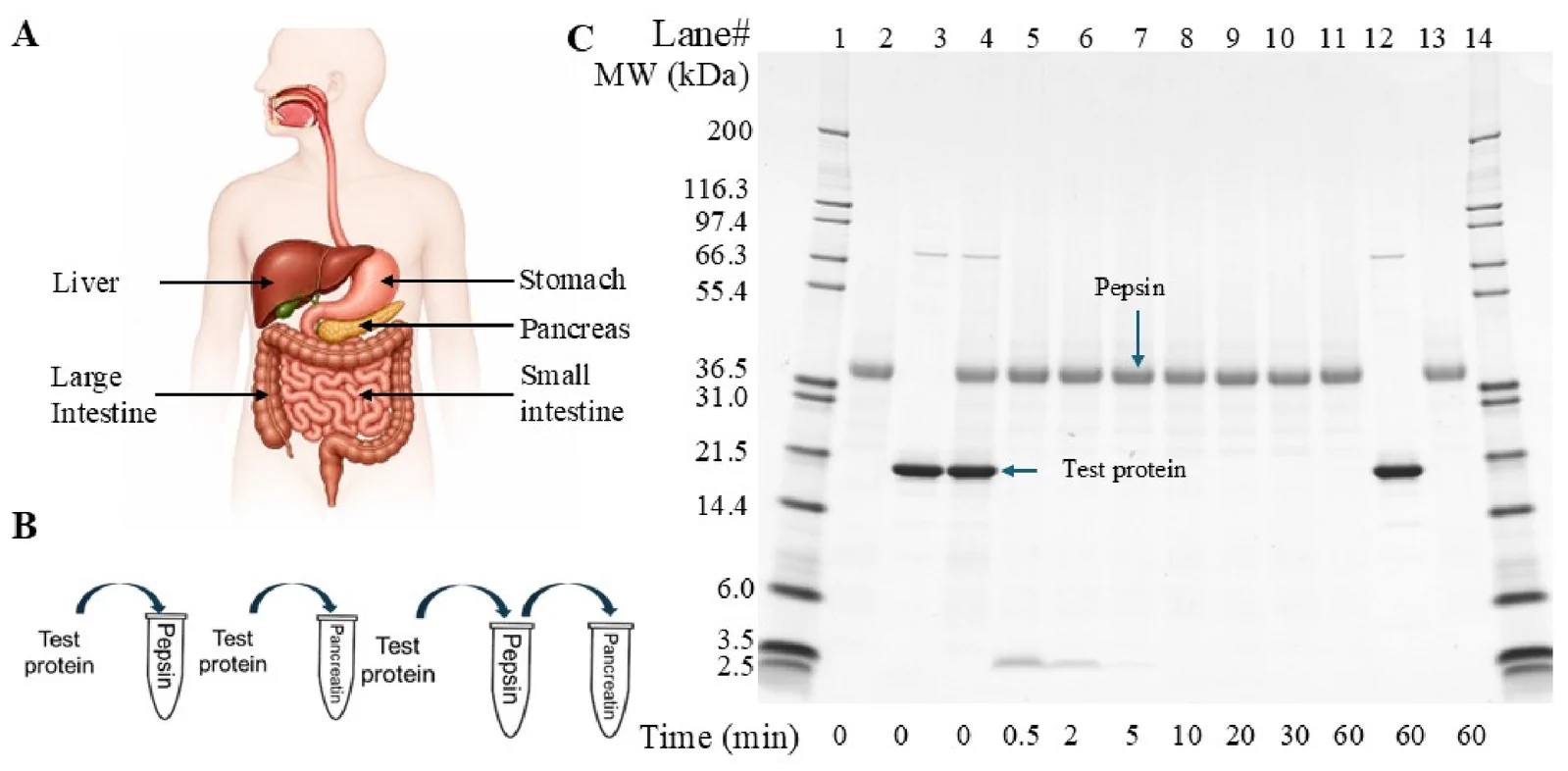
In vitro protein digestibility assessment. (**A**) Schematic representation of human gastrointestinal digestion, in which proteins are sequentially exposed to gastric conditions in the stomach (e.g., low pH and pepsin) followed by intestinal conditions in the small intestine, where pancreatic enzymes (e.g., pancreatin) further digest residual protein. (**B**) In vitro digestion assay used to simulate gastric and intestinal digestion through pepsin, pancreatin, or sequential pepsin–pancreatin treatments under controlled laboratory conditions. (**C**) Representative analysis of protein digestion, where susceptibility to pepsin (and, when applicable, subsequent pancreatin) is evaluated over time. Protein degradation is monitored by SDS–PAGE or immunoblotting, demonstrating the progressive loss of the intact protein and, in some cases, the transient appearance of digestion fragments.

**Figure 2:**
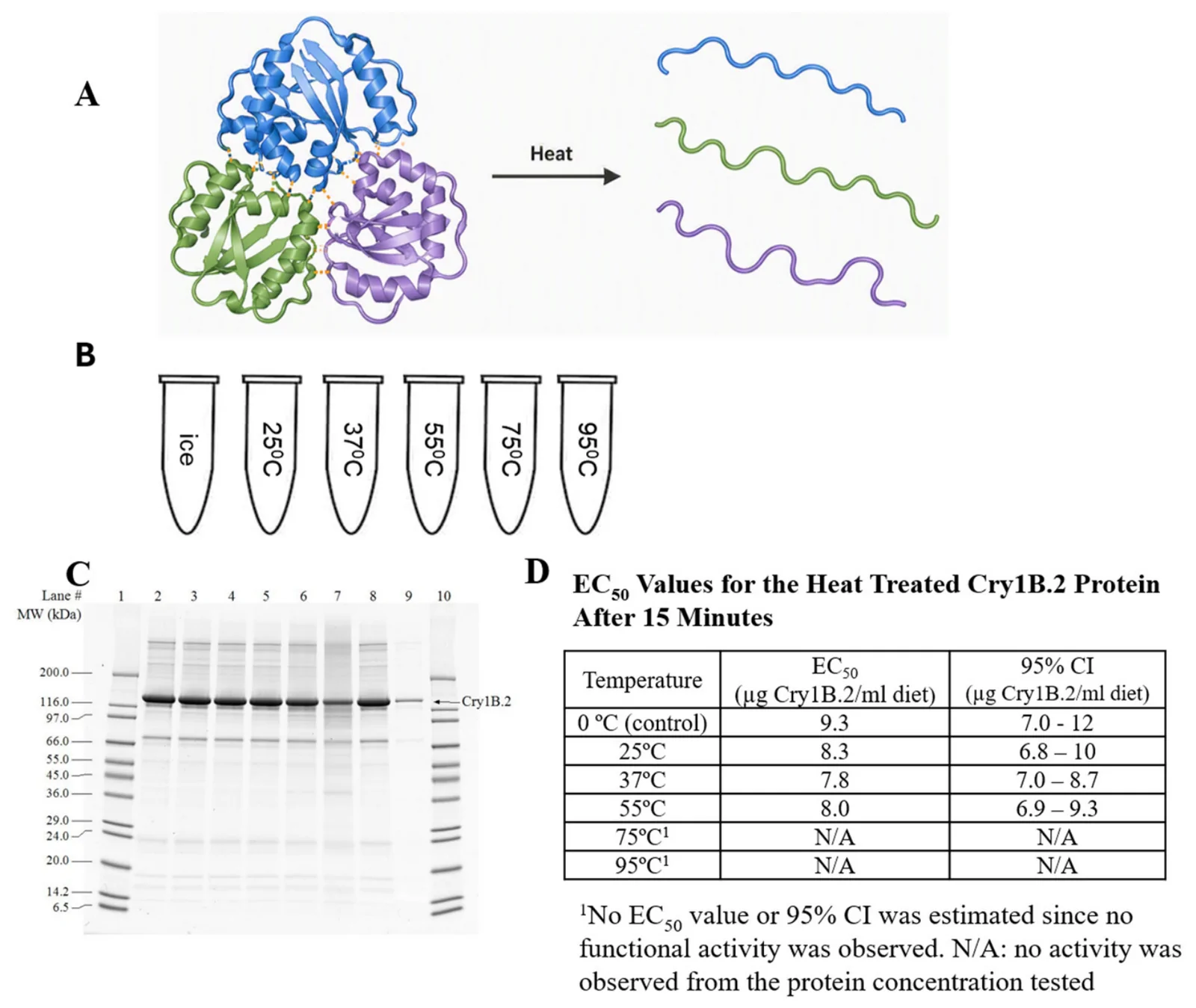
Protein heat stability assessment. (**A**) Schematic illustration of the protein in its native folded conformation, stabilized by intra- and intermolecular interactions, and its transition to an unfolded/denatured state upon heat exposure. Different colors are used to distinguish the individual protein subunits. (**B**) Diagram of the experimental design showing thermal treatment of the test protein across a range of temperatures under controlled conditions to assess heat stability. (**C**) Representative SDS–PAGE analysis of the protein following heat treatment, where reduced band intensity or the appearance of lower molecular weight fragments indicates heat-induced degradation or loss of structural integrity. Lane 1 and 10: molecular weight standards. Lane 2: Cry1B.2 protein control without heat treatment. Lanes 3–7: Cry1B.2 protein heat-treated at 25 °C, 37 °C, 55 °C, 75 °C, and 95 °C, respectively. Lanes 8 and 9: Cry1B.2 reference standards representing 100% and 10% equivalence, respectively. (**D**) Representative functional assay demonstrating loss of biological activity of the Cry three domain protein following heat treatment. EC_50_: Half maximal effective concentration on growth inhibition. CI: confidence interval.

**Figure 3:**
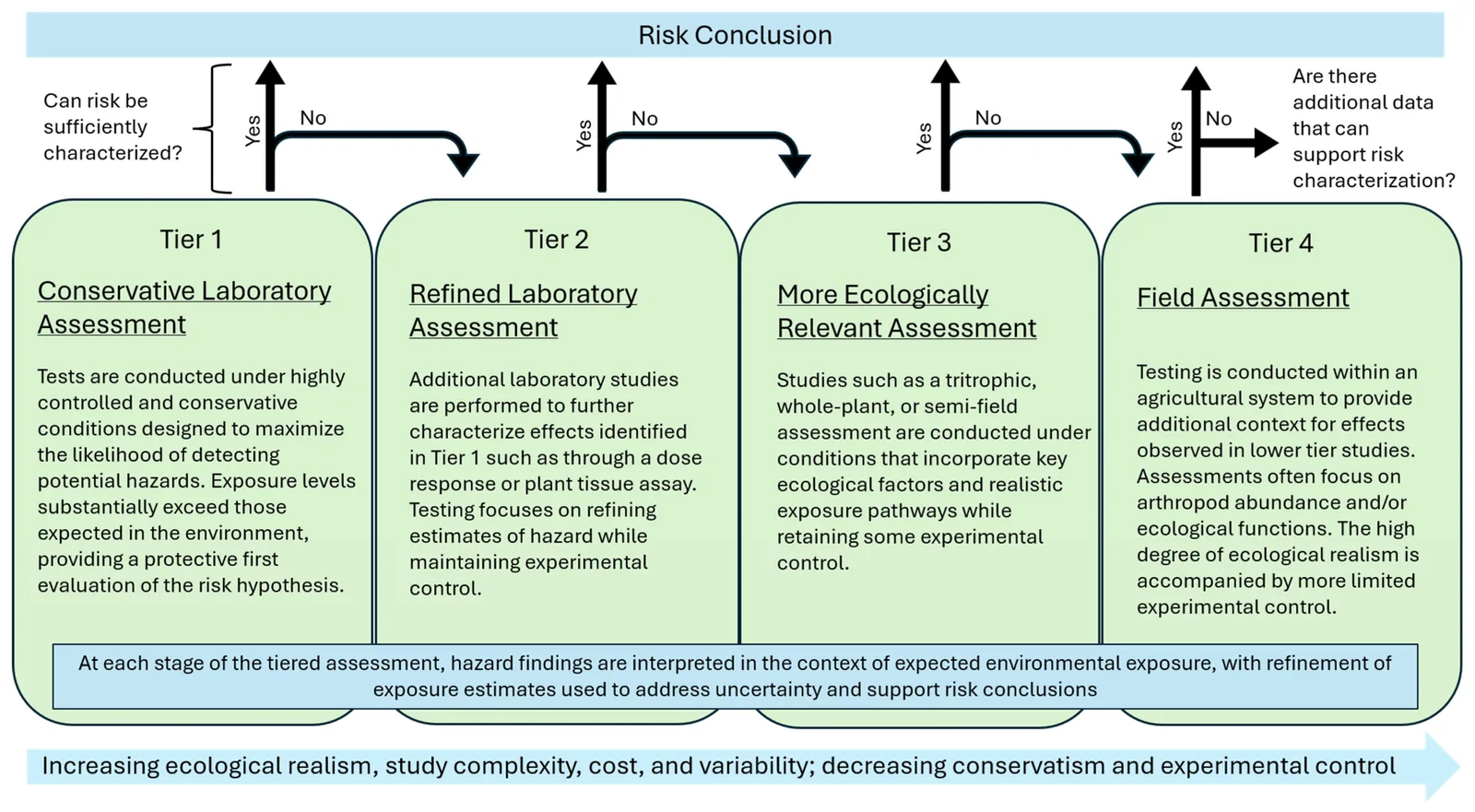
The tiered testing approach to non-target organism testing. Adapted from [12,14].

**Figure 4:**
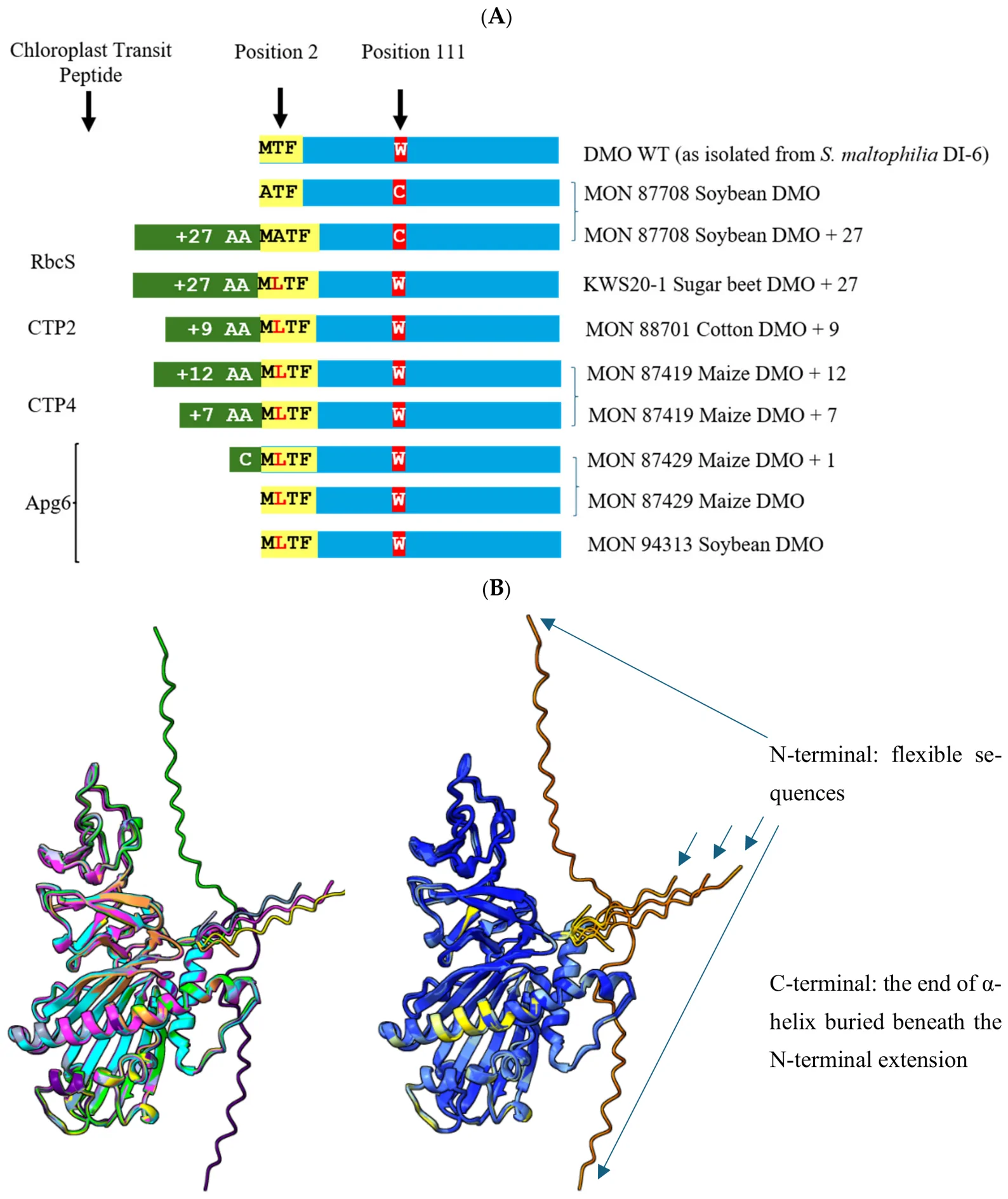
DMO variants expressed in GM crops and structural comparison. (**A**) Amino acid sequence comparison of DMO variants expressed in GM crops relative to the wild-type (WT) DMO derived from *Stenotrophomonas maltophilia*. Numbers and residues shown at the N-terminus indicate additional amino acids introduced during incomplete CTP processing. Arrows indicate amino acid differences relative to the WT sequence. **MON 87708**: Two processed forms are present: (i) a mature form following complete removal of the CTP and intervening sequence, and (ii) a form retaining a 27-amino-acid N-terminal extension derived from the pea Rubisco small subunit (RbcS) transit peptide and intervening sequence. Additional modifications include an alanine at position 2 and a substitution of cysteine for tryptophan at position 111 (relative to the WT DMO sequence). **KWS20-1 sugar beet**: A single form containing the same 27-amino-acid RbcS-derived N-terminal extension as observed for one form of DMO in MON 87708, but tryptophan at position 111. **MON 88701**: Contains nine additional N-terminal amino acids derived from the *Arabidopsis thaliana* 5-enolpyruvylshikimate-3-phosphate synthase (EPSPS; CTP2) transit peptide, along with a leucine at position 2. **MON 87419**: Comprises a mixture of forms with 7 and 12 additional N-terminal amino acids derived from a petunia EPSPS (CTP4) transit peptide, together with a leucine substitution. **MON 87429**: Includes a form with an additional N-terminal cysteine arising from incomplete processing of the *Arabidopsis thaliana* Albino and Pale Green 6 (Apg6)-derived CTP, as well as a completely processed mature form equivalent to that expressed in MON 94313. **MON 94313:** Amino acid sequence identical to WT DMO except for a leucine at position 2. (**B**) Superposition of DMO variant structures. Crop DMO variant sequences were in silico folded using AlphaFold2, ranked0.pdb models were superimposed to the DMO WT and rendered as ribbons. Left panel shows crop DMO variants with varying N-terminal lengths: DMO WT (cyan), MON 87708 Soybean (gray), MON 87708 Soybean + 27 (dark purple), KWS20-1 Sugar beet DMO + 27 (green), MON 88701 Cotton DMO + 9 (magenta), MON 87419 Maize DMO + 12 (yellow), MON 87419 Maize DMO + 7 (steel-blue), MON 87429 Maize DMO + 1 (orange) and MON 94313 Soybean DMO (periwinkle). Right panel shows these crop DMO variants colored by their pLDDT confidence measure, with blue representing high confidence, yellow indicating the backbone is expected to be modeled well, and orange indicating low confidence.

**Figure 5:**
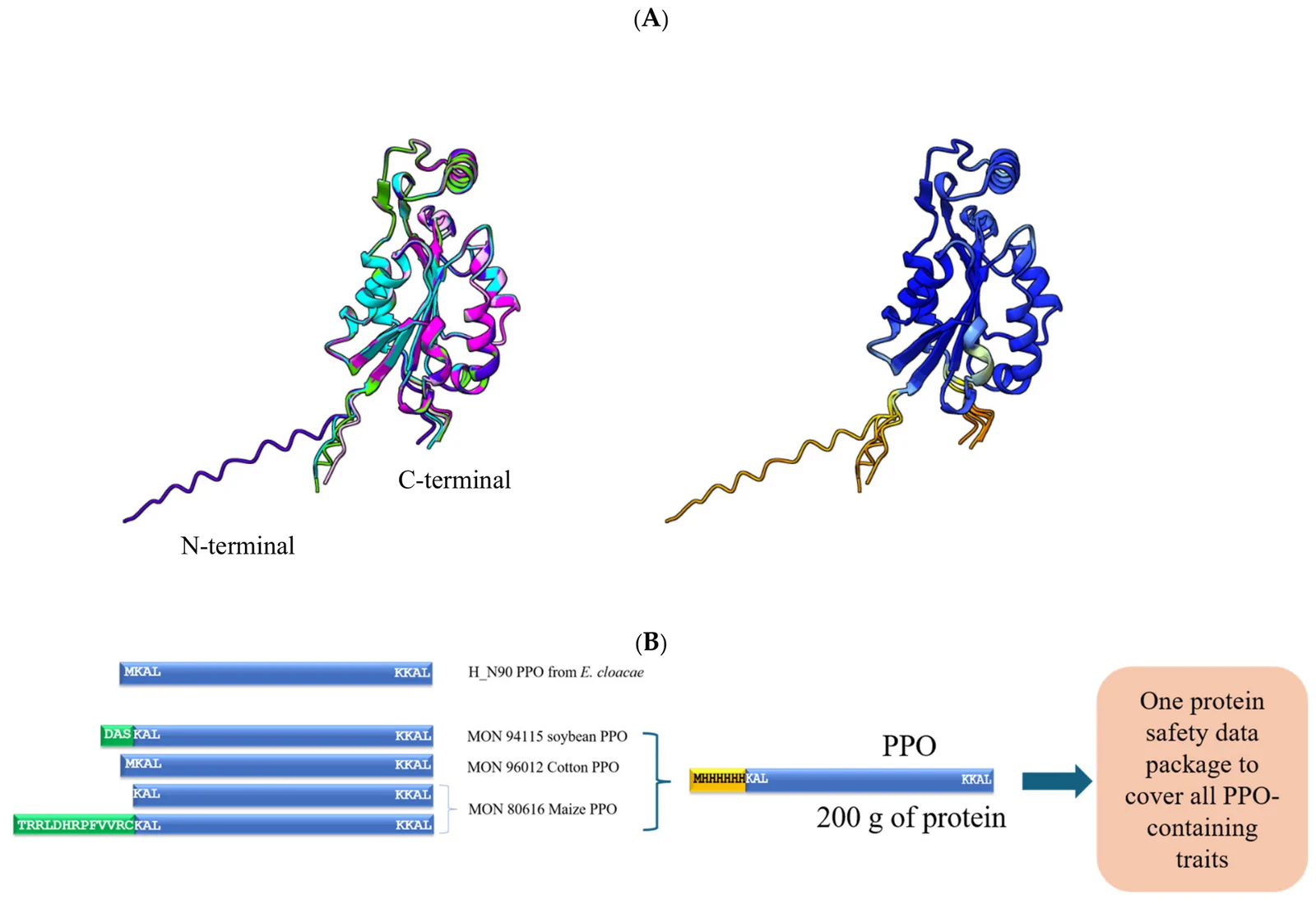
Structural comparison of PPO variants and overview of the bridging strategy supporting safety assessment. (**A**) Superposition of PPO variant structures. Left panel shows multiple in-silico folded (AlphaFold2) crop PPO variants overlayed as ribbons using UCSF ChimeraX Matchmaker: Cotton PPO (cyan), Maize PPO (purple), PPO (light pink), Soy PPO (lime green), and tag-free PPO (magenta). Right panel displays these crop PPOs colored by their pLDDT confidence measure, with blue representing high confidence, yellow indicating the backbone is expected to be modeled well, and orange indicating low confidence (N- and C- termini). (**B**) Bridging strategy. Schematic representation of PPO variants derived from GM soybean, cotton, and maize, alongside the recombinant *E. coli*–produced PPO used as the primary test material. Sequence differences reflect variation in CTP processing. In soybean, incomplete processing of the *Adansonia digitata* Apg6-derived CTP results in the retention of three additional amino acids. In cotton, the *Arabidopsis thaliana* Apg6 CTP is completely processed, with retention of a methionine residue at the junction between the CTP and the mature PPO. In maize, fusion with an *Arabidopsis thaliana* Apg6 sequence results in two variants: a completely processed form and a variant retaining 13 additional N-terminal amino acids. Following demonstration of equivalence among these PPO variants and between plant- and microbe-produced PPO, a single safety assessment package generated using *E. coli*–produced PPO supports the regulatory submission of all PPO-containing traits.

**Figure 6:**
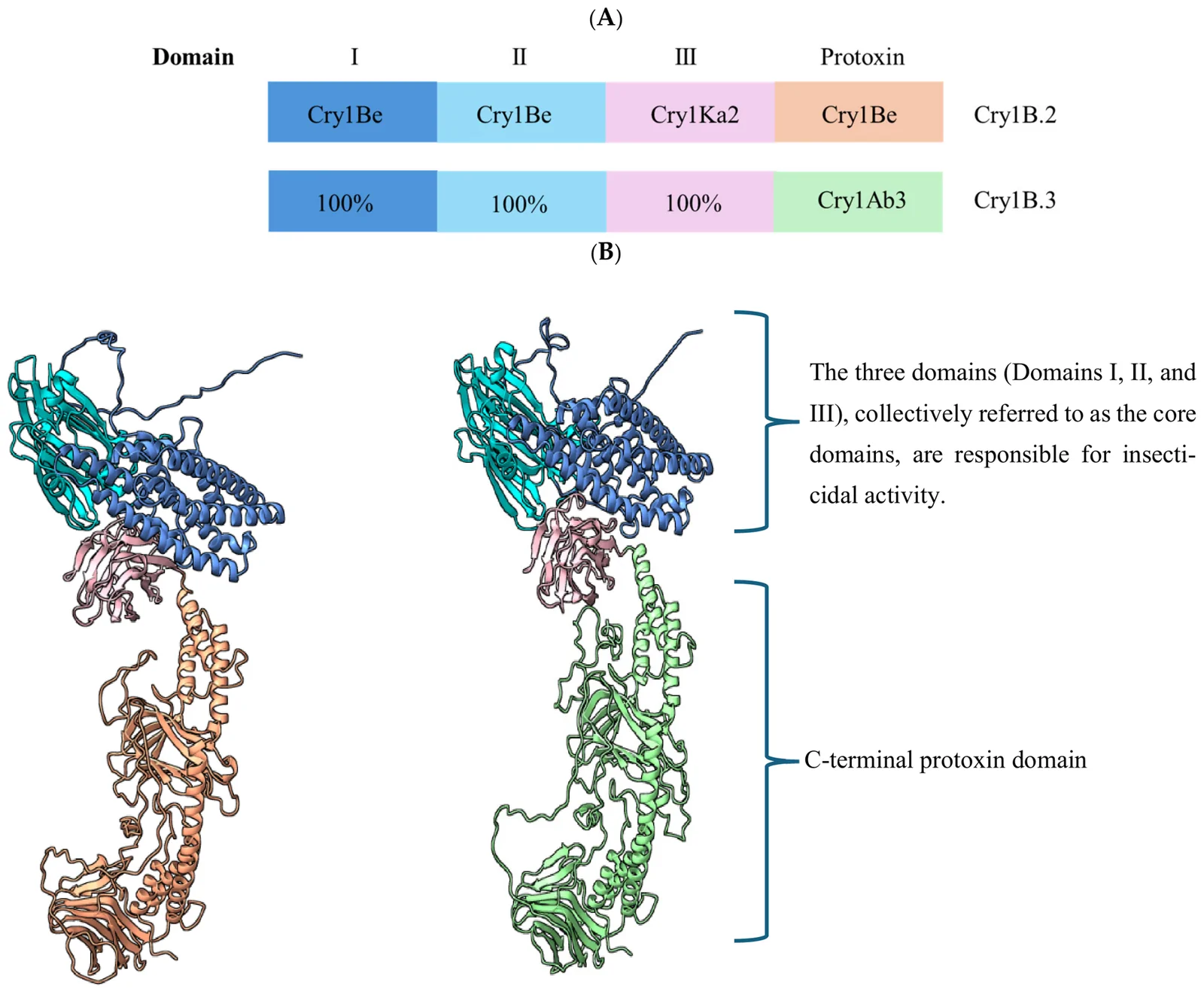
Cry1B.2 and Cry1B.3 sequence and structural comparison. (**A**) Schematic representation of the primary domain architecture of Cry1B.2 and Cry1B.3 proteins. Distinct color shades are used to indicate the origin of individual domains. For clarity, the lengths of the domains shown are not proportional to the actual amino acid lengths of the corresponding domains. (**B**) Cry1B.2 (left) and Cry1B.3 (right) in-silico folded (AlphaFold2) structures are displayed as ribbons with color scheme as in Figure 6A (DI in blue, DII in cyan, DIII in pink, Cry1B.2 C-terminal protoxin in beige versus Cry1B.3 C-terminal protoxin in green). The two proteins exhibited a low RMSD of 0.607 Å and TM-score average of 0.862, indicating high structural similarity (Supplementary Table S2).

**Figure 7:**
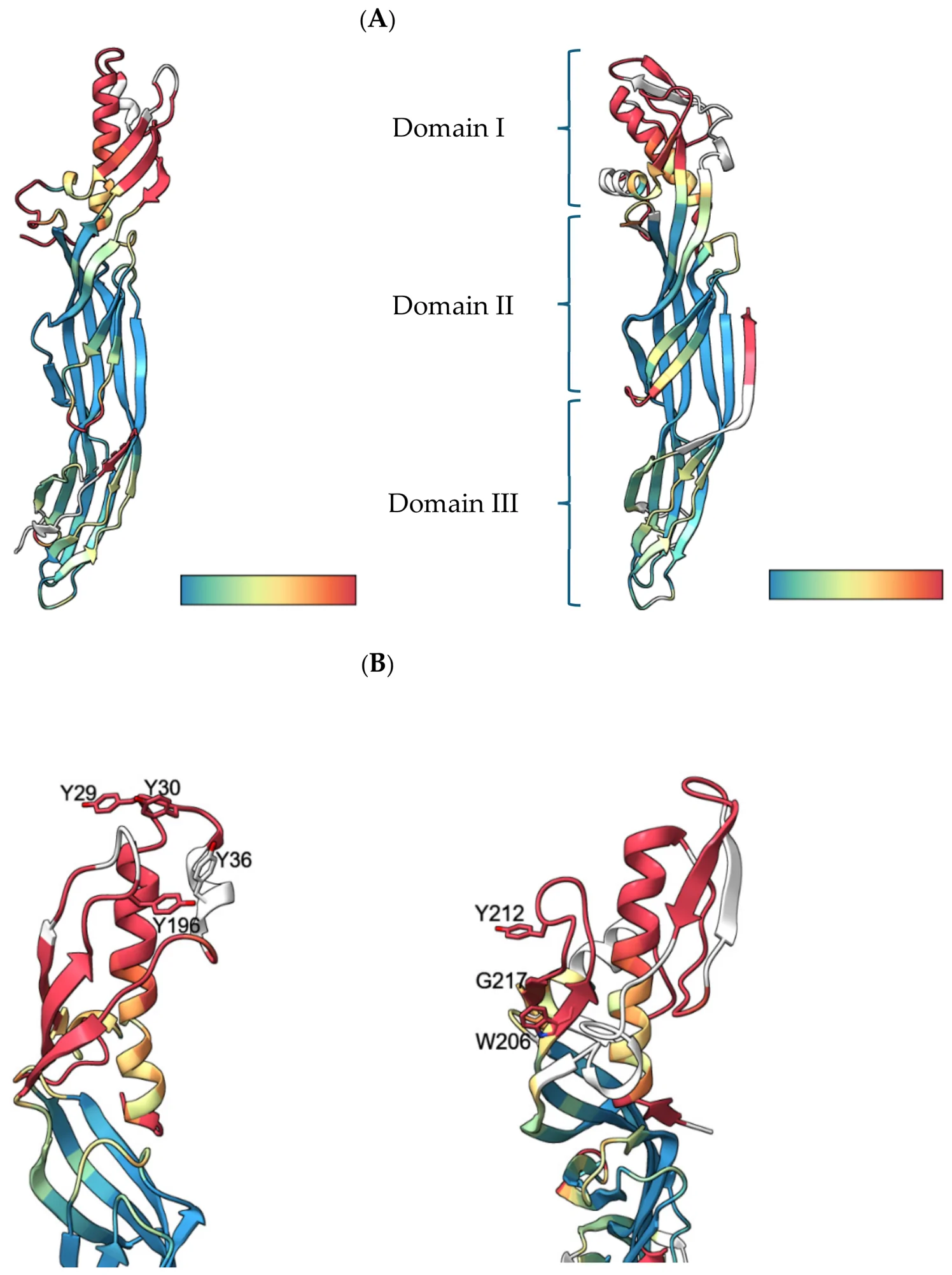
Structure comparison between Epsilon toxin and Mpp75Aa1.1. (**A**) Experimentally determined structures of *C. perfringens* epsilon toxin (1UYJ pdb, left) and *B. laterosporus* Mpp75Aa1.1 (7ML9 pdb, right) were superimposed in UCSF ChimeraX and Cα RMSD values calculated using UCSF ChimeraX Matchmaker—the RMSDs are shown in ribbon from low (1 Å, blue) to high (8 Å, red), with corresponding scale at bottom. White indicates that RMSD was not calculated for those residues. (**B**) Displaying in sticks the key proposed receptor binding residues in high Ca-RMSD Domain I for Epsilon toxin (left) and Mpp75Aa1.1 (right), as in Kouadio et al. [101].

**Table 1:** Safety assessment of DMO variants.

Trait/Event	HoSU	Bioinformatics (Toxins/Allergens)	Characterization and Equivalence ^1^	Heat Stability ^2^	In Vitro Digestibility ^3^	Animal Dietary Exposure ^4^	Human Dietary Exposure ^5^	Acute Toxicity ^6^	Ecological Assessment	Reference
MON 87708 soybean DMO	DMO derived from *Stenotrophomonas maltophilia*; no well-documented HoSU. MON 87708 globally approved in 2011	No biologically relevant sequence similarity to known toxins, allergens or anti-nutritional factors	Catalyzes dicamba demethylation with high substrate specificity; no activity toward endogenous plant substrates. MON 87708 DMO is expressed at relatively high levels and was purified from soybean seed, demonstrating functional activity, recognition by DMO-specific antibodies, and absence of glycosylation. This plant produced DMO was used for safety study.	Functional activity rapidly lost at ≥55 °C	Rapidly degraded by pepsin (≤30 s) and pancreatin (~5 min)	Exceedingly low animal dietary exposure	Exceedingly low human dietary exposure	No treatment-related adverse effects were observed at the limit dose	NA ^7^	[80]
KWS20-1 sugar beet DMO	All DMO variants exhibited characteristics consistent with the established HoSU for DMO in MON 87708 soybean.	All DMO variants show no biologically relevant sequence similarity to known toxins, allergens or anti-nutritional factors, consistent with the MON 87708 DMO.	All DMO variants exhibit conserved structure and enzymatic function, with equivalent catalytic activity toward dicamba. Each plant-produced DMO and its corresponding microbe-produced counterpart demonstrated equivalent molecular weight, immunoreactivity, and functional activity, with no evidence of glycosylation.	All DMO variants exhibit comparable heat lability to MON 87708 soybean DMO.	All DMO variants exhibit rapid degradation under gastrointestinal enzymes, consistent with the MON 87708 dataset.	Exceedingly low animal dietary exposure	Exceedingly low human dietary exposure	No treatment-related adverse effects were observed at the limit dose	NA ^5^	[21,80]
MON 88701 cotton DMO										
MON 87419 maize DMO										
MON 87429 maize DMO										
MON 94313 soybean DMO										

^1^ All DMO variants produced in microbial systems were demonstrated to be physicochemically and functionally equivalent to their respective plant-produced counterparts and were used for safety assessment, except for MON 87708 DMO, for which the plant-produced protein was directly used. ^2,3^ Heat stability and in vitro digestibility are components of the weight-of-evidence assessment and should not be interpreted as standalone predictors of allergenicity. ^4^ <0.01% of total daily dietary protein intake. Maximum exposure calculated with conservative estimations. Exposure is not anticipated to impact animal health. ^5^ Maximum exposure calculated with conservative estimations. Exposure is not anticipated to impact human health. ^6^ There were no hazards identified in the core weight-of-evidence assessment. Therefore, an acute toxicity study is not scientifically justified for hazard characterization. The acute toxicity study was conducted at the request of regulators. ^7^ In the context of herbicide tolerant traits, non-target organism testing is typically not conducted due to lack of plausible risk scenarios to non-target organisms. NA: not applicable.

**Table 2:** Summary of Case Studies in Safety Assessment.

Trait/Event	HoSU	Bioinformatics (Toxins/Allergens)	Characterization and Equivalence	Heat Stability ^1^	In Vitro Digestibility ^2^	Animal Dietary Exposure ^3^	Human Dietary Exposure ^4^	Acute Toxicity ^5^	Ecological Assessment	Reference
MON 96012/MON 80616/MON 94115 PPO	Protoporphyrinogen IX oxidase (PPO; H_N90) from *Enterobacter cloacae*; no documented HoSU	No biologically relevant sequence similarity to known toxins, allergens or anti-nutritional factors	Catalyzes the oxidation of protoporphyrinogen IX to protoporphyrin IX in chlorophyll and heme biosynthesis. Both plant- and microbe-produced PPO show comparable immunoreactivity and functional activity, with no glycosylation. Sequence variants arising from incomplete CTP processing do not affect the enzymatic structure or function. Accordingly, the microbe-produced His-tag PPO was accepted as a surrogate for safety assessment of PPO variants in GM crops	Functional activity lost after 15 min at ≥55 °C	Rapidly digested by pepsin within 2 min and pancreatin within 5 min	Exceedingly low animal dietary exposure	Exceedingly low human dietary exposure	No treatment-related adverse effects were observed at the limit dose	NA ^6^	[28,85]
MON 94637 Cry1B.2/MON 89151 Cry1B.3	Chimeric Cry proteins derived from *Bacillus thuringiensis* (Bt) with established HoSU from parent proteins	No biologically relevant sequence similarity to known toxins, allergens or anti-nutritional factors	Cry1B.2 comprises domains I–II from Cry1Be, domain III from Cry1Ka2, and protoxin domain from Cry1Ab3; Cry1B.3 shares domains I–III but differs in protoxin domain which is from Cry1Be. Both plant-produced Cry1B.2 and microbial-produced Cry1B.2, as well as plant- and microbial-produced Cry1B.3, exhibit comparable molecular weight, immunoreactivity, and functional activity, with no glycosylation. The microbe-produced Cry1B.2 or Cry1B.3 was accepted as a surrogate for safety assessment of Cry1B.2 or Cry1B.3 expressed in the GM crop. Cry1B.2 and Cry1B.3 show similar activity against fall armyworm, corn earworm, and European corn borer in diet-incorporation bioassays.	Functional activity lost after 15 min at ≥75 °C	Rapid degradation by pepsin within 0.5 min; transient ~4 kDa fragment further degraded with pancreatin	Exceedingly low animal dietary exposure	Exceedingly low human dietary exposure	No treatment-related adverse effects were observed at the limit dose	Cry1B.2-Cry1B.3 data bridging supported negligible risk to NTOs, with no additional ecological testing required	[23,86,87,88]
MON 95275 Mpp75Aa1.1	Mpp75Aa1.1 from *Brevibacillus laterosporus* with limited HoSU	Shares limited similarity with ETX/MTX2 protein family; no biologically relevant matches to allergens or anti-nutritional factors	Member of the ETX/MTX2 family of β-pore-forming proteins. Both plant- and microbe-produced Mpp75Aa1.1 show equivalent molecular weight, immunoreactivity and functional activity, with no glycosylation. The microbe-produced Mpp75Aa1.1 was accepted as a surrogate for safety assessment of Mpp75Aa1.1 in GM crops	Functional activity lost after 15 min at ≥55 °C	Rapidly degraded by pepsin (<0.5 min)	Exceedingly low animal dietary exposure	Exceedingly low human dietary exposure	No treatment-related adverse effects were observed at the limit dose	Example ERA for non-traditional source organism with reduced familiarity and expanded biological activity. Demonstrates that the current tiered framework remains fit-for-purpose for evaluating increasingly novel proteins and supporting robust risk conclusions.	[86,87]
MON 89151 Vip3Cb1	Vip3Cb1 from *Paenibacillus popilliae* clade with no HoSU	No biologically relevant similarity to known toxins, allergens or anti-nutritional factors	Structural and functional properties consistent with other Vip3 family proteins, including conserved mode of action. Both plant- and microbe-produced Vip3Cb1 show equivalent molecular weight, immunoreactivity and functional activity, with no glycosylation. The microbe-produced Vip3Cb1 was accepted as a surrogate for safety assessment of Vip3Cb1 in GM crops	Functional activity lost after 15 min at ≥55 °C	Full-length protein degraded within 0.5 and 5 min in pepsin and pancreatin, respectively; transient 8 to 10 kDa fragments observed	Exceedingly low animal dietary exposure	Exceedingly low human dietary exposure	No treatment-related adverse effects were observed at the limit dose	Vip3Cb1 ERA demonstrated negligible risk to NTOs and exemplified a fit-for-purpose data generation	[23,88]

^1,2^ Heat stability and in vitro digestibility are components of the weight-of-evidence assessment and should not be interpreted as standalone predictors of allergenicity. ^3^ <0.01% of total daily dietary protein intake. Maximum exposure calculated with conservative estimations. Exposure is not anticipated to impact animal health. ^4^ Maximum exposure calculated with conservative estimations. Exposure is not anticipated to impact human health. ^5^ There were no hazards identified in the core weight-of-evidence assessment. Therefore, an acute toxicity study is not scientifically justified for hazard characterization. The acute toxicity study was conducted at the request of regulators. ^6^ In the context of herbicide tolerant traits, ERA is not conducted due to lack of plausible risk scenarios to non-target organisms. NA: not applicable.

## Data Availability

All data supporting the findings of this study are available within the article and Supplementary Materials.

## Supplementary Materials

The following supporting information can be downloaded at: https://www.mdpi.com/article/10.3390/plants15213343/s1. Figure S1. Superposition of full-length Cry1 proteins; Table S1. Comparison of DMO Protein Amino Acid Sequence Identity, Root Mean Square Deviation (RMSD), and TM-Score; Table S2. Comparison of Cry Protein Amino Acid Sequence Identity, Root Mean Square Deviation (RMSD), and TM-Score.
