# Supplementary material for: Safety Assessment of Newly Expressed Proteins in Genetically Modified Crops: A Decade of Regulatory Case Studies
Source: Plants (Basel). 2026 Oct 28;15(21):3343. doi: 10.3390/plants15213343 (PMC13765890; doi:10.3390/plants15213343)
Supplement: Supplementary file 1 [file plants-15-03343-s001.zip › plants-4564570-supplementary.pdf]

## Supplementary Materials

**Table S1. Comparison of DMO Protein Amino Acid Sequence Identity, Root Mean Square Deviation (RMSD), and TM-Score**

Values in each cell are presented as Sequence Identity (%, top) / RMSD (Å, middle) / TM-score average (bottom). Amino acid sequence identity of DMO variants was calculated using CLC Genomics Workbench 23. Structural similarity was assessed by pairwise alignment of DMO variant *in silico*–folded structures (AlphaFold2). RMSD values (Å) were determined using UCSF ChimeraX MatchMaker. TM-scores were calculated for the corresponding structures, with possible scores ranging from 0 to 1, with 1 indicating identical structures, and the TM-score reported for compared DMO variants were normalized by average length of chains. RMSD measures structural deviation, whereas TM-score assesses overall structural similarity; lower RMSD values and higher TM-scores indicate greater similarity. Low RMSD values (< 1 Å) and high TM-scores (> 0.9) demonstrate a high degree of structural conservation among DMO variants.

| **Sample** | **ID** | **1** | **2** | **3** | **4** | **5** | **6** | **7** | **8** | **9** |
|---|---|---|---|---|---|---|---|---|---|---|
| **DMO WT** | **1** | — | 99.41 / 0.424 / 0.996 | 92.10 / 0.553 / 0.955 | 92.37 / 0.502 / 0.956 | 94.00 / 0.702 / 0.969 | 95.75 / 0.548 / 0.974 | 98.83 / 0.597 / 0.980 | 97.13 / 0.492 / 0.991 | 99.12 / 0.564 / 0.991 |
| **MON 87708 Soy DMO+0** | **2** | 99.41 / 0.424 / 0.996 | — | 92.64 / 0.474 / 0.957 | 92.10 / 0.283 / 0.957 | 93.71 / 0.673 / 0.970 | 95.47 / 0.489 / 0.976 | 98.54 / 0.516 / 0.982 | 96.84 / 0.492 / 0.991 | 98.83 / 0.493 / 0.992 |
| **MON 87708 Soy DMO+27** | **3** | 92.10 / 0.553 / 0.955 | 92.64 / 0.474 / 0.957 | — | 99.46 / 0.234 / 0.973 | 89.40 / 0.502 / 0.945 | 92.37 / 0.137 / 0.954 | 92.10 / 0.150 / 0.958 | 92.10 / 0.230 / 0.963 | 92.10 / 0.111 / 0.962 |
| **KWS20-1 Sugar beet DMO+27** | **4** | 92.37 / 0.502 / 0.956 | 92.10 / 0.283 / 0.957 | 99.46 / 0.234 / 0.973 | — | 89.95 / 0.529 / 0.946 | 92.92 / 0.446 / 0.955 | 92.64 / 0.448 / 0.958 | 92.64 / 0.177 / 0.964 | 92.64 / 0.173 / 0.962 |
| **MON 88701 Cotton DMO+9** | **5** | 94.00 / 0.702 / 0.969 | 93.71 / 0.673 / 0.970 | 89.40 / 0.502 / 0.945 | 89.95 / 0.529 / 0.946 | — | 94.65 / 0.560 / 0.977 | 94.86 / 0.509 / 0.984 | 96.00 / 0.507 / 0.975 | 94.57 / 0.515 / 0.974 |
| **MON 87419 Maize DMO+12** | **6** | 95.75 / 0.548 / 0.974 | 95.47 / 0.489 / 0.976 | 92.37 / 0.137 / 0.954 | 92.92 / 0.446 / 0.955 | 94.65 / 0.560 / 0.977 | — | 96.88 / 0.460 / 0.987 | 98.58 / 0.212 / 0.983 | 96.59 / 0.107 / 0.982 |

| | | | | | | | | | | |
|---|---|---|---|---|---|---|---|---|---|---|
| **MON 87419 maize DMO+7** | **7** | 98.83 / 0.597 / 0.980 | 98.54 / 0.516 / 0.982 | 92.10 / 0.150 / 0.958 | 92.64 / 0.448 / 0.958 | 94.86 / 0.509 / 0.984 | 96.88 / 0.460 / 0.987 | — | 98.27 / 0.230 / 0.988 | 99.71 / 0.169 / 0.989 |
| **MON 87429 maize DMO+1** | **8** | 97.13 / 0.492 / 0.991 | 96.84 / 0.492 / 0.991 | 92.10 / 0.230 / 0.963 | 92.64 / 0.177 / 0.964 | 96.00 / 0.507 / 0.975 | 98.58 / 0.212 / 0.983 | 98.27 / 0.230 / 0.988 | — | 97.98 / 0.192 / 0.996 |
| **MON 94313 Soybean DMO** | **9** | 99.12 / 0.564 / 0.991 | 98.83 / 0.493 / 0.992 | 92.10 / 0.111 / 0.962 | 92.64 / 0.173 / 0.962 | 94.57 / 0.515 / 0.974 | 96.59 / 0.107 / 0.982 | 99.71 / 0.169 / 0.989 | 97.98 / 0.192 / 0.996 | — |

**Table S2. Comparison of Cry Protein Amino Acid Sequence Identity, Root Mean Square Deviation (RMSD), and TM-Score**

Values in each cell are presented as Sequence Identity (%, top) / RMSD (Å, middle) / TM-score average (bottom). Amino acid sequence identity of Cry proteins was calculated using CLC Genomics Workbench 23. Structural similarity was assessed by pairwise alignment of Cry variant *in silico*–folded structures (AlphaFold2). RMSD values (Å) were determined using UCSF ChimeraX MatchMaker. TM-scores were calculated for the corresponding structures, with possible scores ranging from 0 to 1, with 1 indicating identical structures, and the TM-score reported for compared Cry1 proteins were normalized by average length of chains.

RMSD measures structural deviation, whereas TM-score assesses overall structural similarity; lower RMSD values and higher TM-scores indicate greater similarity. Low RMSD values (< 1 Å) and high TM-scores (> 0.8) demonstrate a high degree of structural conservation among Cry1A variants.

| Sample | ID | 1 | 2 | 3 | 4 | 5 | 6 | 7 |
|---|---|---|---|---|---|---|---|---|
| **Cry1Ab (ABV91087.1)** | 1 | — | 89.36 /<br>0.965<br>0.959 | 82.39 /<br>0.933<br>0.954 | 74.55 /<br>0.530<br>0.866 | 67.79 /<br>0.887<br>0.921 | 59.55 /<br>0.527<br>0.839 | 54.35 /<br>0.697<br>0.895 |
| **Cry1Ac (AAU87037.1)** | 2 | 89.36 /<br>0.965<br>0.959 | — | 92.13 /<br>0.281<br>0.987 | 84.18 /<br>0.772<br>0.914 | 68.91 /<br>1.083<br>0.943 | 59.76 /<br>0.514<br>0.878 | 54.63 /<br>1.143<br>0.911 |
| **Cry1A.105 CAS91272.1** | 3 | 82.39 /<br>0.933<br>0.954 | 92.13 /<br>0.281<br>0.987 | — | 84.83 /<br>0.767<br>0.913 | 70.47 /<br>1.091<br>0.945 | 65.05 /<br>0.467<br>0.871 | 59.73 /<br>1.099<br>0.907 |
| **Cry1A.2 UWS13094.1** | 4 | 74.55 /<br>0.530<br>0.866 | 84.18 /<br>0.772<br>0.914 | 84.83 /<br>0.767<br>0.913 | — | 66.72 /<br>0.785<br>0.910 | 59.71 /<br>0.789<br>0.878 | 54.40 /<br>0.922<br>0.856 |
| **Cry1Da_7 QHN69613.1** | 5 | 67.79 /<br>0.887<br>0.921 | 68.91 /<br>1.083<br>0.943 | 70.47 /<br>1.091<br>0.945 | 66.72 /<br>0.785<br>0.910 | — | 59.08 /<br>0.598<br>0.875 | 54.46 /<br>1.202<br>0.893 |
| **Cry1B.2 UWS13095.1** | 6 | 59.55 /<br>0.527<br>0.839 | 59.76 /<br>0.514<br>0.878 | 65.05 /<br>0.467<br>0.871 | 59.71 /<br>0.789<br>0.878 | 59.08 /<br>0.598<br>0.875 | — | 87.46 /<br>0.607<br>0.862 |

| | | | | | | | | |
|---|---|---|---|---|---|---|---|---|
| **Cry1B.3*** | 7 | 54.35 / 0.697 0.895 | 54.63 / 1.143 0.911 | 59.73 / 1.099 0.907 | 54.40 / 0.922 0.856 | 54.46 / 1.202 0.893 | 87.46 / 0.607 0.862 | — |

* Cry1B.3 shares Domains I, II, and III with Cry1B.2 and contains a C-terminal protoxin domain derived from Cry1Ab3.

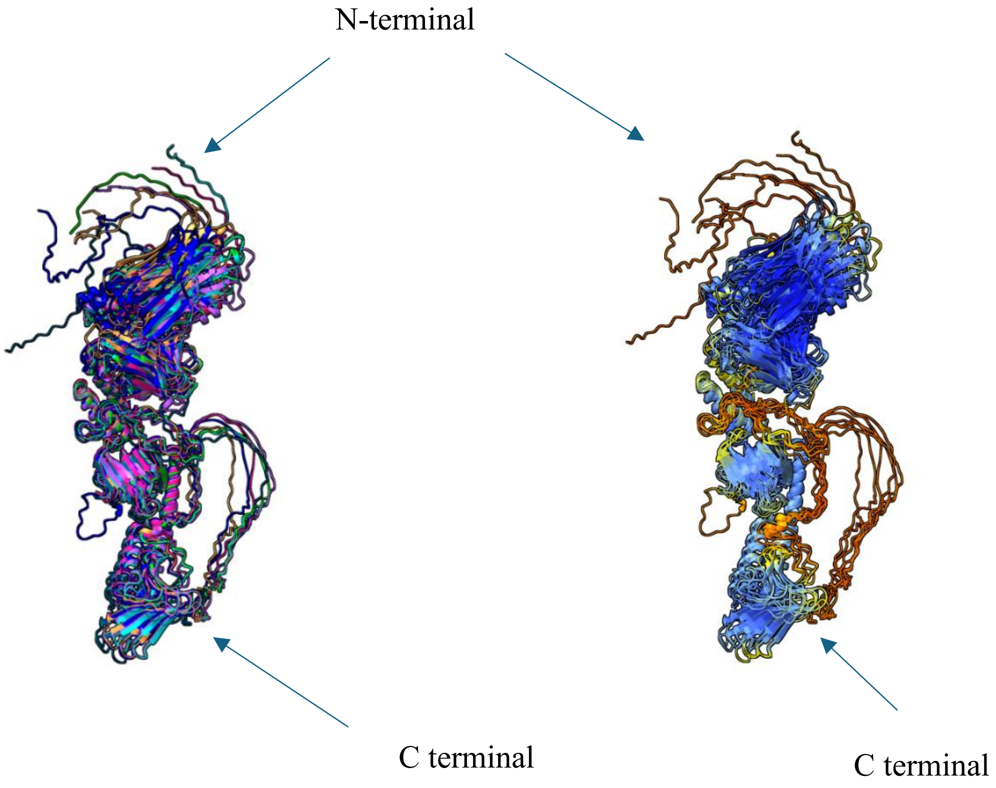


**Figure S1. Superposition of full-length Cry1 proteins**

Left panel shows multiple *in-silico* folded (AlphaFold2) Cry1 protoxins overlaid as ribbons using UCSF ChimeraX Matchmaker: Cry1A.2 (steel blue), Cry1A.105 (cyan), Cry1Ab (purple), Cry1Ac (lime green), Cry1B.2 (orange) Cry1B.3 (dark blue) and Cry1Da_7 (magenta). Right panel displays these Cry1 protoxins colored by their pLDDT confidence measure, with blue representing high confidence, yellow indicating the backbone is expected to be modeled well, and orange indicating low confidence.
